# Rescue of male infertility by human *PRSS55* in transgenic mice establishes a contraceptive research model

**DOI:** 10.1038/s41598-025-09604-9

**Published:** 2025-08-05

**Authors:** Courtney M. Sutton, Kohei Umezu, Daisuke Mashiko, Masahito Ikawa, Irina V. Larina, Thomas X. Garcia, Martin M. Matzuk

**Affiliations:** 1 Center for Drug Discovery, Baylor College of Medicine, Houston, TX USA; 2 Department of Pathology and Immunology, Baylor College of Medicine, Houston, TX USA; 3 Department of Integrative Physiology, Baylor College of Medicine, Houston, TX USA; 4 Department of Experimental Genome Research, Research Institute for Microbial Diseases, Osaka University, Suita, Osaka Japan; 5 The Institute of Medical Science, The University of Tokyo, Minato-ku, Tokyo, Japan; 6 Scott Department of Urology, Baylor College of Medicine, Houston, TX USA; 7 Verna and Marrs McLean Department of Biochemistry and Molecular Biology, Baylor College of Medicine, Houston, TX USA

**Keywords:** Reproductive biology, Cell biology, Molecular biology

## Abstract

The development of non-hormonal male contraceptives requires validated preclinical models. This study investigated whether human orthologs of two mouse testis-specific serine proteases, PRSS55 and TMPRSS12, both essential for male fertility in mice, could functionally rescue the infertility phenotypes of their respective knockout mouse lines. We generated transgenic mouse lines expressing human *PRSS55* with either an extracellularly or intracellularly positioned C-terminal 3xFLAG tag (RES GPI or RES TM), and a line expressing human *TMPRSS12* with a C-terminal 3xFLAG tag (T12 RES), all on their respective mouse null backgrounds. Fertility was assessed through continuous mating trials, and sperm parameters were evaluated. Both *hPRSS55* rescue lines sired offspring; RES GPI males exhibited partially restored fertility with significantly fewer pups per litter compared to controls, while RES TM males demonstrated fertility comparable to controls. In contrast, T12 RES males remained infertile, exhibiting severe defects in sperm motility and other parameters, despite confirmed transgene mRNA expression. These findings indicate that human PRSS55, particularly when tagged intracellularly, can functionally replace its mouse counterpart, validating the RES TM line as a promising model for testing human PRSS55-targeted contraceptives. The inability of *hTMPRSS12* to rescue fertility highlights challenges potentially due to sequence divergence or unconfirmed protein expression levels.

**Supplementary Information:**

The online version contains supplementary material available at 10.1038/s41598-025-09604-9.

## Introduction

The development of safe, effective, and reversible contraceptive options remains a global health priority, with a persistent imbalance in the availability and burden of use between sexes^1,2^. For decades, the contraceptive market has predominantly catered to women, offering a range of hormonal and non-hormonal methods, from oral pills to long-acting reversible contraceptives such as intrauterine devices^1^. In contrast, options for male contraception have remained limited, with condoms and vasectomy being the primary recognized methods^1^. The pursuit of novel male contraceptives has been hampered by challenges associated with hormonal approaches; systemic androgen suppression, while potentially effective, can lead to undesirable physiological consequences, including adverse effects on libido, mood, cardiovascular health, and testicular function, making such strategies less feasible for widespread use^3^. Consequently, there is a pressing need for non-hormonal male contraceptive targets that can provide reversible infertility with minimal systemic side effects.

A promising strategy for developing non-hormonal male contraceptives involves targeting proteins that are specifically expressed in the male reproductive tract and are essential for fertility. Inhibiting such proteins is hypothesized to induce a contraceptive effect with high specificity and a lower risk of off-target effects. Among the candidates for such targeted intervention are various testis-specific and sperm-specific enzymes, including serine proteases, which play critical roles in sperm development, maturation, and function within the female reproductive tract.

Previous research has identified several serine proteases as vital for male reproduction. Notably, transmembrane serine protease 12 (*TMPRSS12*) and serine protease 55 (*PRSS55*) are predominantly expressed in sperm and have been shown to be indispensable for male fertility in mouse models^4–9^. Genetic ablation of either *Tmprss12* or *Prss55* in mice results in male infertility, primarily due to the inability of sperm to successfully navigate the uterotubal junction (UTJ) and reach the site of fertilization^4–9^. Furthermore, PRSS55 deficiency has been correlated with defects in the processing or localization of ADAM3 (a disintegrin and metalloproteinase domain 3), another protein essential for sperm migration and fertilization, suggesting PRSS55 may act upstream in a cascade critical for these processes^8–10^. While the precise molecular mechanisms are still under investigation, the infertility phenotypes associated with *Tmprss12* and *Prss55* knockouts highlight their potential as targets for male contraception.

Despite the clear importance of these proteases in mouse fertility, a critical step towards validating them as targets for human contraception is to determine if their human orthologs can functionally substitute for the mouse proteins. Establishing humanized mouse models—wherein the mouse gene is replaced or complemented by its human counterpart—would provide an invaluable preclinical platform for evaluating the efficacy and specificity of contraceptive agents designed against the human proteins. However, the functional interchangeability of these specific human and mouse reproductive proteases has not been fully established. Therefore, this study aimed to investigate the efficacy of human *PRSS55* and human *TMPRSS12* in rescuing the infertility phenotypes of their respective knockout mouse lines. Specifically, we sought to generate transgenic mouse lines expressing human *PRSS55* (*hPRSS55*) with two different epitope tag configurations (extracellular vs. intracellular) on a mouse *Prss55* null background; generate a transgenic mouse line expressing human *TMPRSS12* (*hTMPRSS12*) on a mouse *Tmprss12* null background; and assess the fertility of these humanized transgenic male mice and characterize key sperm parameters. Successfully rescuing fertility with the human transgenes would validate these humanized mouse lines as suitable models for in vivo testing of contraceptive compounds targeting human PRSS55 or TMPRSS12.

This paper describes the generation of humanized transgenic mouse lines, reports on their sperm characteristics, and details the outcomes of comprehensive fertility trials. The findings provide insights into the functional conservation of these critical reproductive proteases and the feasibility of using such humanized models for future contraceptive research and indicates that the reproductive processes through which these proteins function are conserved.

## Materials and methods

### Ethics statement

All animal procedures were conducted in strict accordance with the National Institutes of Health (NIH) guidelines and received approval from the Institutional Animal Care and Use Committee (IACUC) at Baylor College of Medicine (BCM) under protocol AN-716. This study was designed and reported in accordance with the ARRIVE (Animal Research: Reporting of In Vivo Experiments) guidelines^11^.

### Animals

Sexually mature male mice were utilized for all phenotypic analyses. In specific experiments, heterozygous (HET) mice served as controls, as prior observations indicated their fertility data were comparable to wild-type (WT) mice. All mice were maintained under a 12-h light/12-h dark cycle with ad libitum access to food and water. *Prss51/55*^+*/d*^ and *Tmprss12*^+*/d*^ mice were originally obtained from Osaka University, Japan, and colonies were subsequently expanded at BCM to generate homozygous knockout (*Prss51/55*^*d/d*^ and *Tmprss12*^*d/d*^) male and female mice.

### Transgene construct design and synthesis

**Fig. 1:**
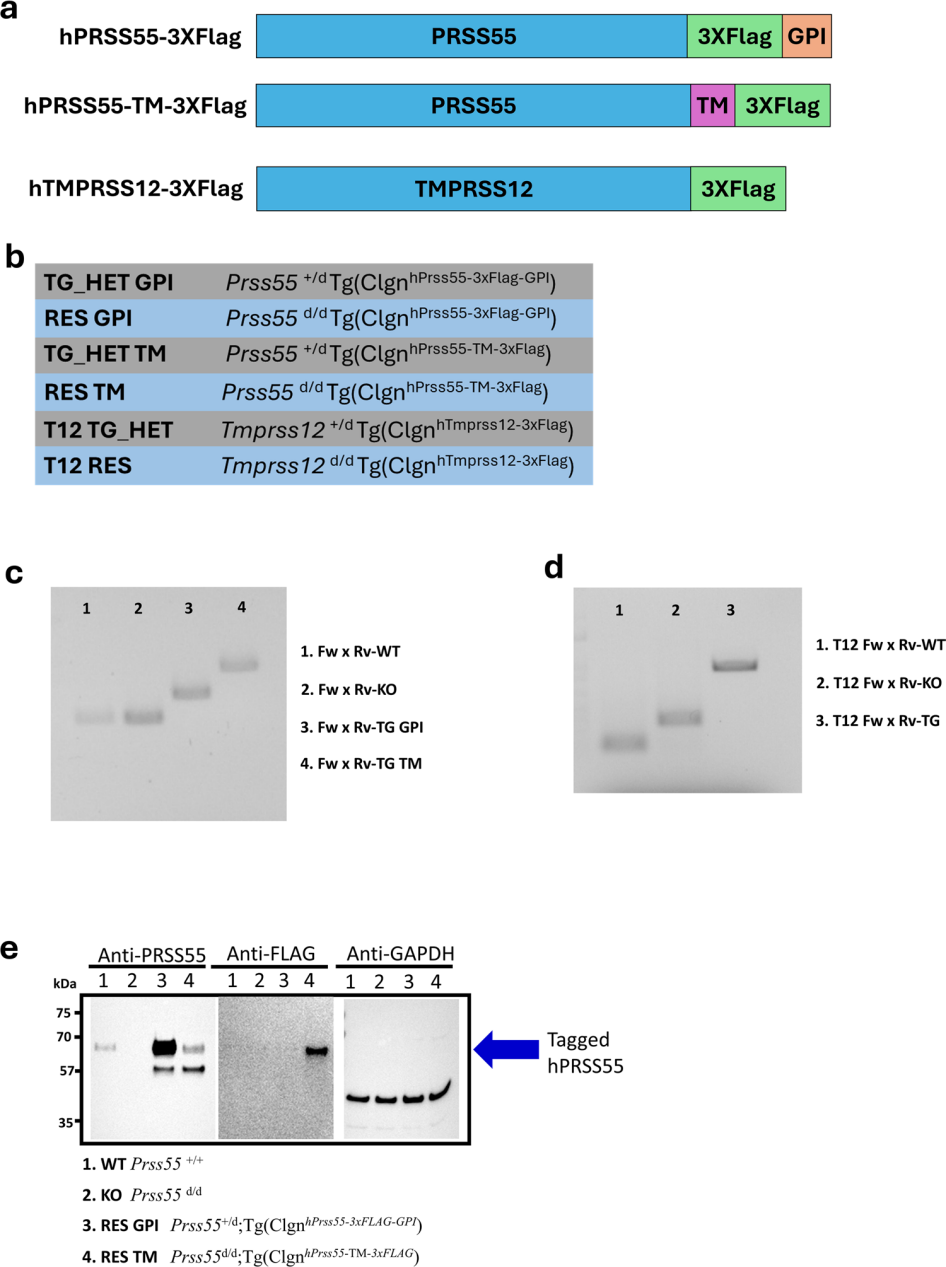
Developing *hPRSS55* and *hTMPRSS12* transgenic mouse line. (**a**) Graphic of the *hPRSS55* with a 3XFlag tag added at the C-terminus of the protein to generate the *PRSS55* transgenic mouse lines. The GPI construct has the Flag tag inserted prior to the GPI anchor making the tag extracellular, while the TM construct has the Flag tag following the transmembrane domain so that flag tag would be located intracellularly. Graphic of the *hTMPRSS12* with a 3XFlag tag added at the C-terminus of the protein to generate the *TMPRSS12* transgenic mouse line. (**b**) Graphic detailing the genotypes of the transgenic mice. (**c**) Genotyping of *PRSS55* alleles. Primers shown in Fig. 1c amplify specific amplicons for the WT (Fw-WT x Rv-WT), KO (Fw-KO x Rv-KO), TG GPI (Fw-TG GPI x Rv-TG GPI), and TG TM (Fw-TG TM x Rv-TG TM) alleles. This gel image was cropped so only 4 wells of the gel are present, the full gel can be found in the Supplementary Fig. S3. (**d**) Genotyping of *TMPRSS12* alleles. Primers shown in Fig. 1d amplify specific amplicons for the T12 WT (Fw-WT x Rv-WT), T12 KO (Fw-KO x Rv-KO) and T12 TG (Fw-TG x Rv-TG) alleles. This gel image was cropped so only 3 wells of the gel are present (lanes 4–6), the full gel can be found in the Supplementary Fig. S1. (**e**) Immunoblot analysis to confirm expression of PRSS55 in HET, KO, RES GPI, and RES TM mice in the first blot. With confirmatory blotting of Anti-FLAG in the transgenic mice in the second blot and Anti-GAPDH in all mice in the third blot. These blots were cropped so only bands between the molecular weight of 75 kDa and 35 kDa are shown, the full blots can be found in the Supplementary Fig. S4.

To generate humanized *PRSS55* transgenic mice, two constructs encoding the human *PRSS55* sequence (RefSeq: NM_198464.4) with a C-terminal 3xFLAG tag were designed. The first construct, designated *hPRSS55-3xFLAG-GPI*, was engineered to retain the native glycosylphosphatidylinositol (GPI) anchor signal sequence, thereby positioning the 3xFLAG tag extracellularly. The second construct, *hPRSS55-TM-3xFLAG*, involved replacing the native GPI anchor signal sequence with a transmembrane domain sequence, followed by the 3xFLAG tag, resulting in an intracellular tag location (Fig. 1a). For the humanized *TMPRSS12* transgenic mice, a construct encoding the human *TMPRSS12* sequence (RefSeq: NM_182559.3) was designed with a 3xFLAG tag inserted immediately following the predicted transmembrane domain (Fig. 1a). All transgene constructs were designed in the Matzuk laboratory, synthesized by Genewiz (Azenta Life Sciences, South Plainfield, NJ, USA), and initially cloned into a pUC vector. For preliminary expression testing, constructs were subcloned into an expression vector containing a CAG promoter and transfected into HEK293 cells to test protein expression at the correct molecular weight via immunoblotting. Subsequently, for spermatid-specific expression in vivo, the CAG promoter was replaced with the mouse Clgn (calmegin) promoter, as previously described^12,13^.

### Generation of transgenic founders and line establishment

The finalized *pCLGN-hPRSS55-3xFLAG-GPI*, *pCLGN-hPRSS55-TM-3xFLAG*, and *pCLGN-hTMPRSS12-3xFLAG* plasmids were linearized and purified. Large-scale purification and pronuclear injection into C57BL/6N zygotes were performed by the Genetically Engineered Rodent Models (GERM) Core at BCM^5,14^. Founder mice carrying the transgenes (Fig. 1a) were identified by PCR genotyping of tail-tip DNA. *Prss55* transgenic founders [*Prss55*^+*/*+^*;Tg(CLGN*^*hPrss55*^*)*] were bred with *Prss55*^+*/d*^ or *Prss55*^*d/d*^ mice to establish distinct lines and subsequently generate experimental cohorts: TG_HET GPI [*Prss55*^+*/d*^*;Tg(CLGN*^*hPrss55-3xFLAG-GPI*^*)*], RES GPI [*Prss55*^*d/d*^*;Tg(CLGN*^*hPrss55-3xFLAG-GPI*^*)*], TG_HET TM [*Prss55* + */d;Tg(CLGN*^*hPrss55-*^*™*^*-3xFLAG*^*)*], and RES TM [*Prss55*^*d/d*^*;Tg(CLGN*^*hPrss55-*^*™*^*-3xFLAG*^*)*] mice (Fig. 1b). Similarly, *Tmprss12* transgenic founders [*Tmprss12*^+*/*+^*;Tg(CLGN*^*hTmprss12-3xFLAG*^*)*] were bred with *Tmprss12*^+*/d*^ or *Tmprss12*^*d/d*^ mice to generate experimental T12 RES [*Tmprss12*^*d/d*^*;Tg(CLGN*^*hTmprss12-3xFLAG*^*)*] mice and corresponding control lines (Fig. 1b). Breeding strategies maintained distinct founder lineages without intercrossing.

### Genotyping

Genomic DNA was extracted from tail snips. PCR was performed to detect the presence of the human *Prss55* transgenes using the following primer pairs: TG GPI Fw-TG: 5′-AGAACTGAGTGTCGTGCTGG-3′ and Rv-TG: 5′-GCCCTTGTCATCGTCATCCT-3′. TG TM Fw-TG: 5′-TTCCGTGGCAGGTGAGTATT-3′ and Rv-TG: 5′-ATCACCGTCATGGTCTTTGT-3′ (Fig. 1c). The human *Tmprss12* transgene was detected using primers: T12 TG Fw-TG: 5′-TTGTGGAACAGCACCGCTTA-3′ and T12 TG Rv-TG: 5′-TCACCGTCATGGTCTTTGTAGT-3′ (Fig. 1d). Genotyping for endogenous mouse *Prss55* alleles (wild-type or knockout) was conducted using primers: WT alleles: Fw-WT: 5′-AAGCTAGCAGCTATTCGGTGGTCAGCAGA-3′ and Rv-WT: 5′-AAGTCGACTCCTGAGCATAGAAGCAGTGG-3′. KO alleles: Fw-KO: 5′-AAGTCGACGAATGAACGGTCTCACGGTT-3′ and Rv-KO: 5′-AAGTCGACGACTCCTTCATAGAGAGGGA-3′ (Fig. 1c). Genotyping for endogenous mouse *Tmprss12* alleles was performed using primers: WT alleles: Fw-WT: 5′-ACCAGACTCTGTTGGGACCT-3′ and Rv-WT: 5′-AAGCTCGGGTTGCTGTAGAC-3′. KO alleles: Fw-KO: 5′-TCTCTCCCATCCATGCCTCA-3′ and Rv-KO: 5′-CAGTGTTGACATGGACGCAC-3′ (Fig. 1d).

### Male fertility assessment

Sexually mature (≥ 6 weeks old) male mice of TG_HET and RES genotypes were continuously housed with individual HET or KO females (as appropriate for the male’s genotype to assess rescue or maintain KO lines) for a period of 4 months. Breeding cages were monitored daily, and the number of pups per litter per male was recorded to determine fertility status and litter size.

### Protein extraction and immunoblot analysis

Testicular germ cells (TGCs) were collected from adult male mice from the *Prss55* lines. Protein extraction was performed as previously detailed by Sutton et al.^5^. Protein lysates were resolved by SDS-PAGE and transferred to nitrocellulose membranes. Membranes were blocked using Bullet Blocking One (Nacalai USA, San Diego, CA, USA). Immunoblotting was performed using primary antibodies against PRSS55 (1:500 dilution; custom monoclonal antibody generated by the Protein and Monoclonal Antibody Core, BCM, Houston, TX, USA), ADAM3 (1:1000 dilution; Santa Cruz Biotechnology, Dallas, TX, USA), FLAG-HRP (1:1000 dilution; Sigma-Aldrich, St. Louis, MO, USA), and GAPDH-HRP (1:5000 dilution; Proteintech, Rosemont, IL, USA). Primary antibody incubations were performed overnight at 4 °C. The anti-PRSS55 antibody was developed at BCM by immunizing female *Prss55*^*d/d*^ mice with recombinant hPRSS55 (Proteos, Kalamazoo, MI, USA), followed by hybridoma generation, screening by ELISA and Western blot, clone selection, expansion, and cryopreservation. Appropriate HRP-conjugated secondary antibodies were used where necessary, and signals were detected using an enhanced chemiluminescence system.

### RNA extraction and reverse transcriptase

Total RNA was extracted from whole testes of adult male mice using the Direct-zol RNA Miniprep kit (Zymo Research, Irvine, CA, USA) according to the manufacturer’s protocol. First-strand cDNA was synthesized from total RNA using the SuperScript III Reverse Transcriptase kit (Invitrogen, Carlsbad, CA, USA) following the manufacturer’s instructions. PCR was performed using cDNA as a template with primers specific for human *TMPRSS12* (Fw: 5′-GTGAGAGAGAGGTGGGTCCT-3′ and Rv: 5′-CAGCCATGTCCGTAACTGGT-3′) to confirm transgene expression in the rescue mice. Mouse small ribosomal subunit protein uS3 (Rps3) (Fw: 5′-TGCTATGGTGTGCTTCGGTT-3′ and Rv: 5′-CAGCTGCCAAGACCCTGTTA-3′) was used as an internal control for cDNA quality and loading (Supplementary Fig. S1).

### Computer-assisted sperm analysis (CASA)

Sperm were extracted from the cauda epididymis of adult male mice into pre-warmed Enhance Sperm Wash medium with Gentamicin (Vitrolife, Gothenburg, Sweden) and maintained at 37 °C throughout the procedure. Following incubation for 15 min (pre-capacitation) or 90 min (post-capacitation/hyperactivation), a 6 µL aliquot of the sperm suspension was loaded into a single chamber of a dual-chambered 20 µm-depth Leja semen analysis slide (Spectrum Technologies, Healdsburg, CA, USA). Sperm motility parameters were measured using the Hamilton Thorne CEROS II system (Hamilton Thorne Ltd., Beverly, MA, USA). Parameters assessed included: sperm count (M/mL), percentage of motile sperm (motility %), percentage of progressively motile cells (progressive cells %), percentage of static cells (static cells %), percentage of hyperactivated cells (hyperactivation %), average path velocity (VAP; µm/sec), curvilinear velocity (VCL; µm/sec), progressive (straight-line) velocity (VSL; µm/sec), mean amplitude of lateral head displacement (ALH; µm), beat cross frequency (BCF; Hz), linear coefficient (LIN = VSL/VCL; %), straightness (STR = VSL/VAP; %), and wobble (WOB = VAP/VCL; %). A detailed description of the CASA procedure can be found in Sutton et al.^5^.

### Sperm waveform analysis

Sperm were extracted from the cauda epididymis by making approximately 30 incisions with dissection scissors in 500 µL of pre-warmed (37 °C) Enhance Sperm Wash medium with Gentamicin (Vitrolife). Sperm suspensions were incubated at 37 °C for 15 and 90 min. For analysis, sperm were placed onto a 100 µm-depth chamber slide maintained at 37 °C on a heating plate. Sperm motility was recorded for over 100 s at 40.2 frames per second using a ZEISS Axio Observer inverted microscope (Carl Zeiss, Oberkochen, Germany) at 20X magnification, equipped with an Axiocam 702 high-speed camera (Carl Zeiss). Video data were exported as consecutive image frames using ZEN 2 Blue Edition software (Zen Blue 3.4.91, https://www.zeiss.com/microscopy/en/products/software/zeiss-zen.html Carl Zeiss). Fourteen consecutive frames displaying in-focus sperm were selected for waveform analysis. Sperm heads and tails were segmented using the Image Segmenter App within MATLAB (The MathWorks Inc., Natick, MA, USA) with custom code. Segmented images were color-coded by time and overlaid to trace the sperm waveform. This analysis was performed on sperm from three WT mice and three KO mice for each protease. Sperm waveform analysis methods were adapted from Nozawa et al.^15^.

### Statistical analysis

Statistical significance for comparisons between two groups was evaluated using the two-tailed unpaired Student’s t-test, assuming unequal variances unless otherwise noted. For comparisons involving more than two groups, such as fertility test results and body/organ weights, a non-parametric Kruskal–Wallis test was performed, followed by Dunn’s multiple comparisons test where appropriate. All data are presented as means ± standard error of the mean (SEM). A *P* value < 0.05 was considered statistically significant. Statistical analyses were performed using Prism version 10.2.2 (GraphPad Software, Boston, MA, USA).

## Results

### Generation and establishment of humanized *PRSS55* and *TMPRSS12* transgenic mouse lines

To investigate the potential for human PRSS55 and TMPRSS12 to rescue infertility in knockout mouse models, transgenic mouse lines expressing human *PRSS55* or human *TMPRSS12* were generated (Supplementary Fig. S8 and S9).

For the humanized *PRSS55* lines, two constructs were utilized: one encoding human *PRSS55* with its native GPI anchor and a C-terminal 3xFLAG tag positioned extracellularly (*hPRSS55-3xFLAG-GPI*), and another where the native GPI anchor was replaced with a transmembrane domain, positioning the C-terminal 3xFLAG tag intracellularly (*hPRSS55-TM-3xFLAG*) (Fig. 1a). Following pronuclear injection by the BCM Genetically Engineered Rodent Models Core, 43 pups were born for the GPI construct, of which five founders carried the *hPRSS55-3xFLAG-GPI* transgene. For the TM construct, 31 pups were delivered, with four founders identified carrying the *hPRSS55-TM-3xFLAG* transgene. Founder mice, with initial genotypes of *Prss55*^+*/*+^*;Tg(CLGN*^*hPrss55-3xFLAG-GPI*^*)* or *Prss55*^+*/*+^*;Tg(CLGN*^*hPrss55-*^*™*^*-3xFLAG*^*)*, were subsequently bred with *Prss55*^+*/d*^ or *Prss55*^*d/d*^ mice to establish distinct transgenic lines. A systematic breeding strategy, avoiding intercrossing of founder lines, was employed to generate male mice for fertility studies with the genotypes *Prss55*^*d/d*^*;Tg(CLGN*^*hPrss55-3xFLAG-GPI*^*)* (RES GPI) and *Prss55*^*d/d*^*;Tg(CLGN*^*hPrss55-*^*™*^*-3xFLAG*^*)* (RES TM).

For the humanized *TMPRSS12* line, a construct encoding human *TMPRSS12* with a 3xFLAG tag inserted after the transmembrane domain was used (Fig. 1a). From the pronuclear injection, 17 pups were delivered, yielding three founder mice carrying the *hTMPRSS12-3xFLAG* transgene. These founders, with an initial genotype of *Tmprss12*^+*/*+^*;Tg(CLGN*^*hTmprss12-3xFLAG*^*)*, were bred with *Tmprss12*^+*/d*^ or *Tmprss12*^*d/d*^ mice. Similar to the *PRSS55* lines, a breeding scheme that avoided intercrossing founder lines was used to obtain male mice with the genotype *Tmprss12*^*d/d*^*;Tg(CLGN*^*hTmprss12-3xFLAG*^*)* (T12 RES) for subsequent fertility assessment. Expression of human *PRSS55* protein in the respective rescue lines was confirmed by immunoblot analysis (Fig. 1e). ADAM3 expression in the testis and epididymis tissue was also looked at in the humanized *PRSS55* lines (Supplementary Fig. S5). ADAM3 was present in all testis samples (WT, KO, RES GPI, RES TM) but expression appeared to drop off in the transgenic lines in the epididymis tissue. Additionally, the presence of PRSS55 in *Adam3* KO mice (Supplementary Fig. S6) was observed by immunoblot analysis, and PRSS55 is present in both the testis and sperm. The presence of *hTmprss12* mRNA in the testes of T12 RES mice was confirmed by RT-PCR (Supplementary Fig. S1) protein expression of *hTmprss12* was not detected from immunoblot analysis (Supplementary Fig. S7).

### Sperm waveform characteristics in *Prss55* and *Tmprss12* knockout mice

**Fig. 2:**
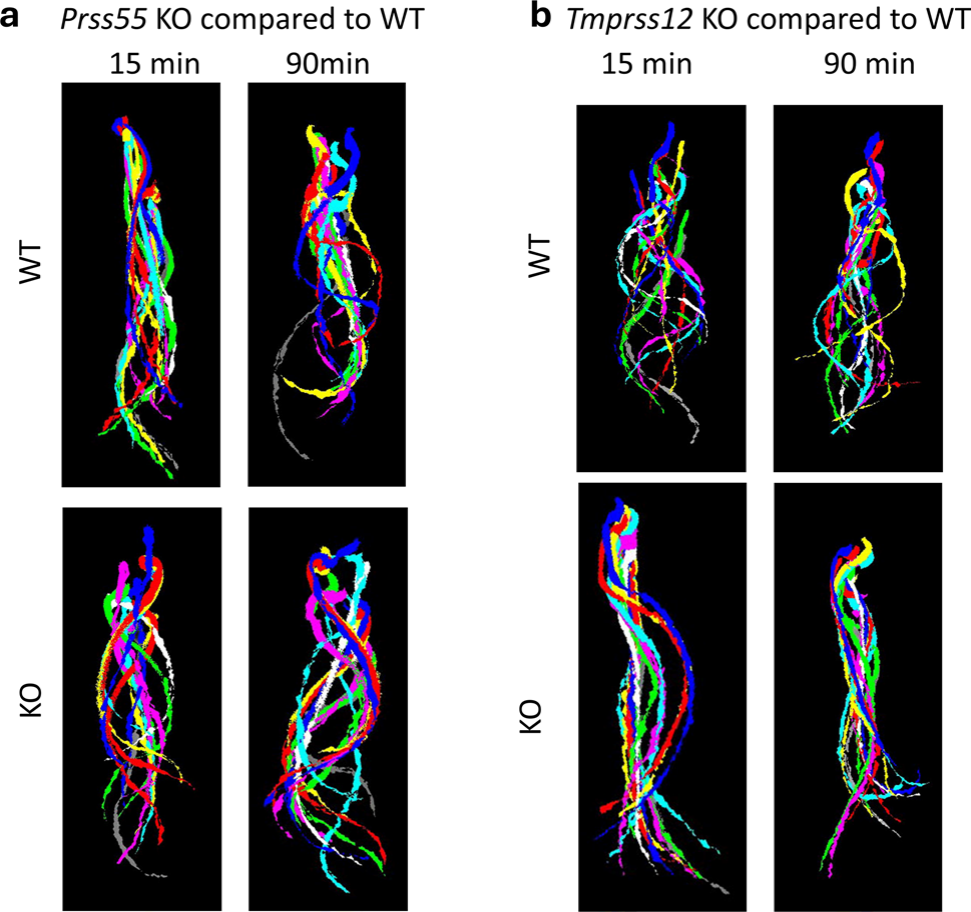
Waveform Analysis of *Prss55* KO mice and *Tmprss12* KO compared to WT mice. (**a**) Comparison of sperm waveform between WT and *Prss55* KO mice at 15- and 90-min show that KO mice appear to have a wider range of flagellar movement at 15 min compared to WT mice. (**b**) Comparison of sperm waveform analysis of WT and *Tmprss12* KO mice at 15- and 90-min show that KO mice have what appears to be a more rigid tail movement than the wider loops of the WT mouse.

To assess potential alterations in sperm flagellar beat patterns resulting from the absence of PRSS55 or TMPRSS12, sperm waveform analysis was performed on samples from adult wild-type (WT) and knockout (KO) male mice. In *Prss55* KO mice, sperm flagella exhibited a wider arch in their beat pattern, particularly evident at the 15-min time point post-extraction, when compared to WT sperm (Fig. 2a). In *Tmprss12* KO mice, sperm flagella displayed beats with a smaller amplitude. When these movements were traced over time, *Tmprss12* KO sperm appeared relatively stagnant compared to the wider, circular flagellar beats observed in WT sperm (Fig. 2b).

### Sperm parameters in humanized *PRSS55* transgenic rescue mice

**Fig. 3:**
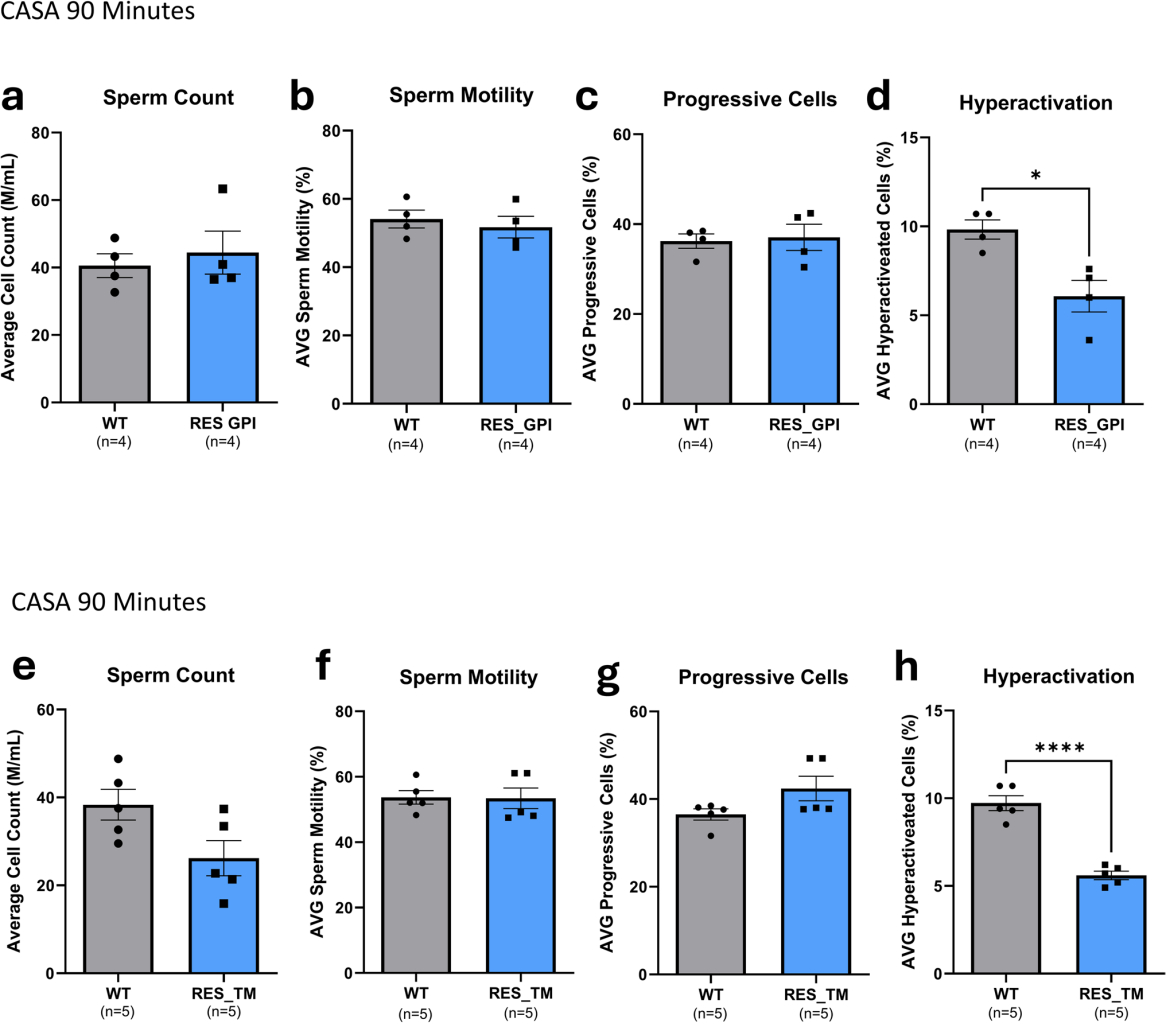
CASA for RES GPI and RES TM mice at 90 min. There was no difference in sperm count (**a**, **e**), sperm motility (**b**, **f**), or progressive cells (**c**, **g**) for RES GPI or RES TM mice at 90 min. Differences were observed in hyperactivation (d, h) for RES GPI (*P* = 0.01) and RES TM (*P* < 0.0001) compared to WT mice at 90 min.

To investigate sperm parameters in the humanized *PRSS55* rescue lines CASA was performed at 15 and 90 min post-extraction, representing pre-capacitation/hyperactivation and post-capacitation/hyperactivation states, respectively (Fig. 3). At the 15-min time point, no significant differences in sperm parameters were observed between WT mice and either the RES GPI or RES TM lines (Supplementary Fig. S2 and S3). At the 90-min time point, sperm from both RES GPI and RES TM lines displayed a statistically significant decrease in the average percentage of hyperactivated cells compared to WT mice (RES GPI: *P* = 0.01; RES TM: *P* < 0.0001) (Fig. 3d,h). Other sperm parameters, including sperm count, overall motility, and progressive motility, were not significantly different between the RES lines and WT mice at this time point (Fig. 3a–c, e–g).

### Sperm parameters in humanized *TMPRSS12* transgenic rescue mice

**Fig. 4:**
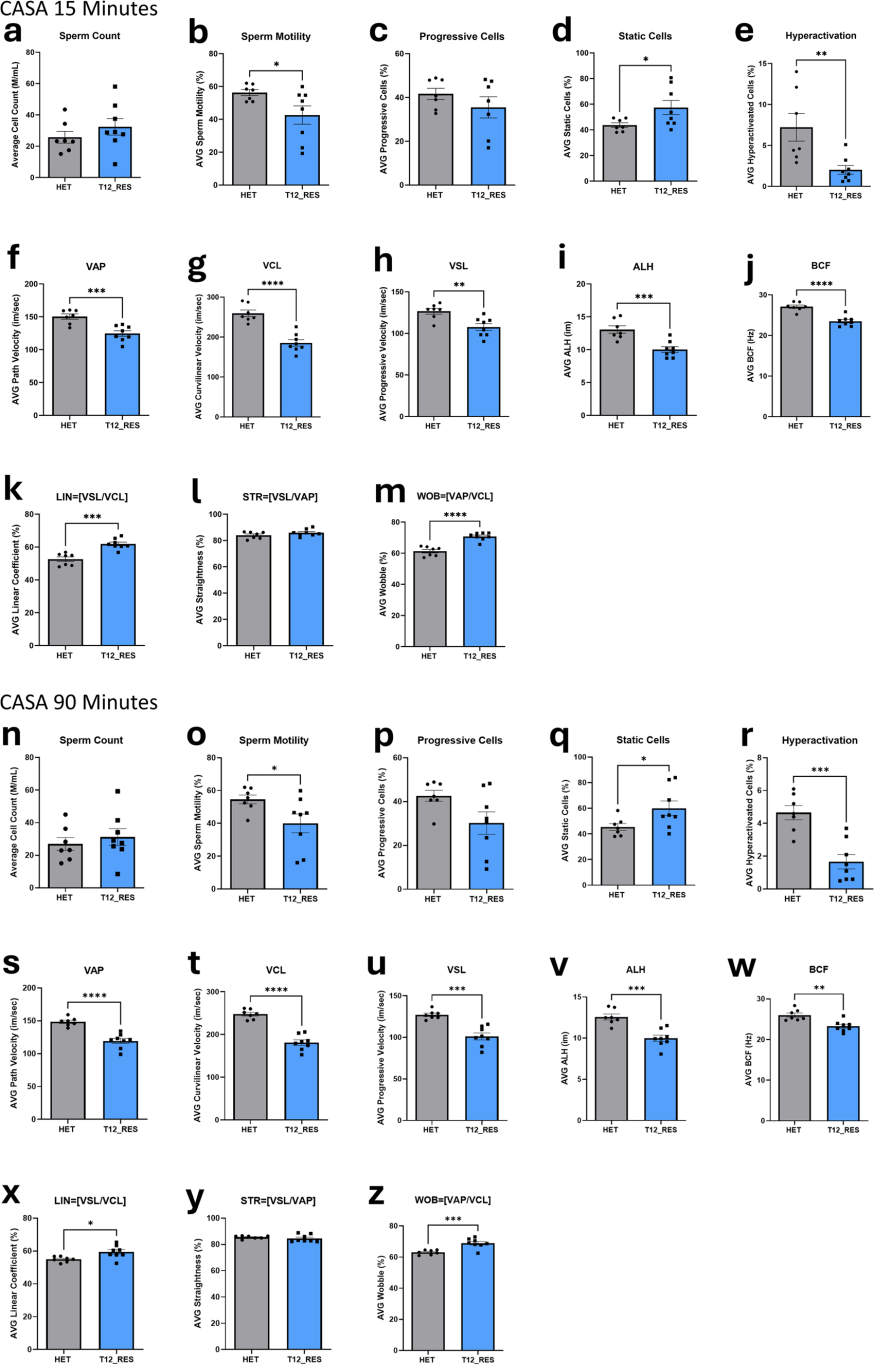
CASA for T12 RES at 15 and 90 min. At 15 min differences were noted in motility (*P* = 0.04) (**b**), static cells (*P* = 0.04) (**d**), hyperactivation (*P* = 0.008) (**e**), VAP (*P* = 0.0007) (**f**), VCL (*P* < 0.0001) (**g**), VSL (*P* = 0.004) (**h**), ALH (*P* = 0.0009) (**i**), BCF (*P* < 0.0001) (**j**), LIN (*P* = 0.0002) (**k**), and WOB (*P* < 0.0001) (**m**). At 90 min T12 RES males differed from HET males in motility (*P* = 0.04) (**o**), static cells (*P* = 0.04) (**q**), hyperactivation (*P* = 0.0003) (**r**), VAP (*P* < 0.0001) (**s**), VCL (*P* < 0.0001) (**t**), VSL (*P* = 0.0001) (**u**), ALH (*P* = 0.0003) (**v**), BCF (*P* = 0.0017) (**w**), LIN (*P* = 0.01) (**x**), and WOB (*P* = 0.0006) (**z**). There were no observed differences in sperm count (**a**,**n**), progressive cells (**c**,**p**), or STR (**l**,**y**), at either time point.

To evaluate sperm parameters in the T12 RES male mice, CASA was conducted at 15 and 90 min post-extraction, with heterozygous (*Tmprss12*^+*/d*^; HET) mice serving as controls (Fig. 4). At the 15-min time point, sperm from T12 RES males exhibited significant decreases in motility (*P* = 0.04), percentage of hyperactivated cells (*P* = 0.008), VAP (*P* = 0.0007), VCL (*P* < 0.0001), VSL (*P* = 0.004), ALH (*P* = 0.0009), and BCF (*P* < 0.0001) when compared to HET controls. Concurrently, significant increases were observed in the percentage of static cells (*P* = 0.04), LIN (*P* = 0.0002), and WOB (*P* < 0.0001) in T12 RES sperm (Fig. 4b,d–m). These differences persisted at the 90-min time point. T12 RES sperm continued to show significant decreases in motility (*P* = 0.04), hyperactivation (*P* = 0.0003), VAP (*P* < 0.0001), VCL (*P* < 0.0001), VSL (*P* = 0.0001), ALH (*P* = 0.0003), and BCF (*P* = 0.0017). Correspondingly, there were significant increases in static cells (*P* = 0.04), LIN (*P* = 0.01), and WOB (*P* = 0.0006) (Fig. 4o,q–z). No significant differences were observed in sperm count, percentage of progressive cells, or STR between T12 RES and HET mice at either the 15-min or 90-min time points (Fig. 4a,c,l,n,p,y).

### Fertility assessment of humanized *PRSS55* transgenic mice

**Fig. 5:**
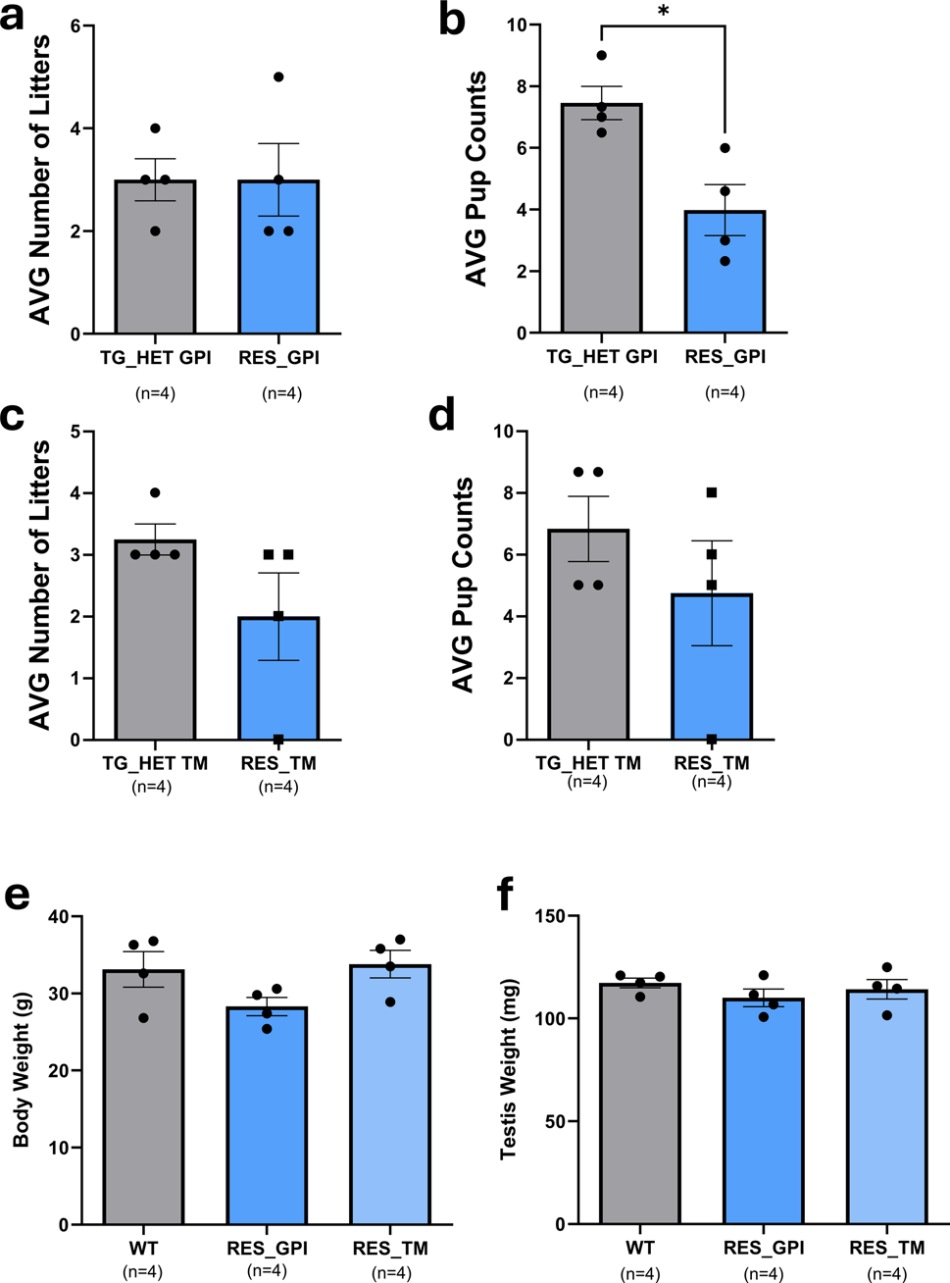
*Prss55* transgenic mice can rescue fertility in *Prss55* KO mice. (**a**) Average pup counts for the number of RES GPI mice differed (*P* = 0.0019) from the TG_HET GPI mice. Litter size was measured by the number of pups born. (**b**) Average litter per male per month, from natural mating of RES GPI mice compared to TG_HET GPI mice did not differ. (**c**) Average pup counts for the number of RES TM mice did not differ from the TG_HET TM mice. Litter size was measured by the number of pups born. (**d**) Average litter per male per month, from natural mating of RES TM mice compared to TG_HET TM mice did not differ. (**e**) The average body weight for RES GPI and RES TM mice did not differ between WT mice. (**f**) The average testis weight for RES TM and RES TM mice did not differ between WT mice.

To determine if transgenic expression of human *PRSS55* could rescue the infertility phenotype of *Prss55* KO mice, mating trials were conducted over a 4-month period. Sexually mature male mice of TG_HET GPI [*Prss55*^+*/d*^*;Tg(CLGN*^*hPrss55-3xFLAG-GPI*^*)*], RES GPI [*Prss55*^*d/d*^*;Tg(CLGN*^*hPrss55-3xFLAG-GPI*^*)*], TG_HET TM [*Prss55* + */d;Tg(CLGN*^*hPrss55-*^*™*^*-3xFLAG*^*)*], and RES TM [*Prss55*^*d/d*^*;Tg(CLGN*^*hPrss55-*^*™*^*-3xFLAG*^*)*] genotypes were continuously housed with *Prss55*^+*/d*^ or *Prss55*^*d/d*^ females, respectively, and litter production was recorded. TG_HET GPI males (N = 4) sired an average of 3.0 ± 0.41 litters per month, with an average of 7.46 ± 0.54 pups per litter (Fig. 5a). RES GPI males (N = 4) sired an average of 3.0 ± 0.70 litters per month; however, the average number of pups per litter was significantly lower at 3.98 ± 0.82 (*P* = 0.01 compared to TG_HET GPI) (Fig. 5b). TG_HET TM males (N = 4) sired an average of 3.25 ± 0.25 litters per month, with an average of 6.83 ± 1.06 pups per litter (Fig. 5c). RES TM males (N = 4) sired an average of 2.0 ± 0.71 litters per month, with an average of 4.75 ± 1.70 pups per litter; these values were not statistically different from their TG_HET TM counterparts (Fig. 5d). No statistically significant differences were observed in body weight (WT: 33.13 ± 2.34 g; RES GPI: 28.30 ± 1.18 g; RES TM: 33.80 ± 1.79 g) or testis weight (WT: 117.3 ± 2.36 mg; RES GPI: 110.05 ± 4.28 mg; RES TM: 114.20 ± 4.79 mg) among WT, RES GPI, and RES TM mice (Fig. 5e,f).

### Fertility assessment of humanized *TMPRSS12* transgenic mice

**Fig. 6:**
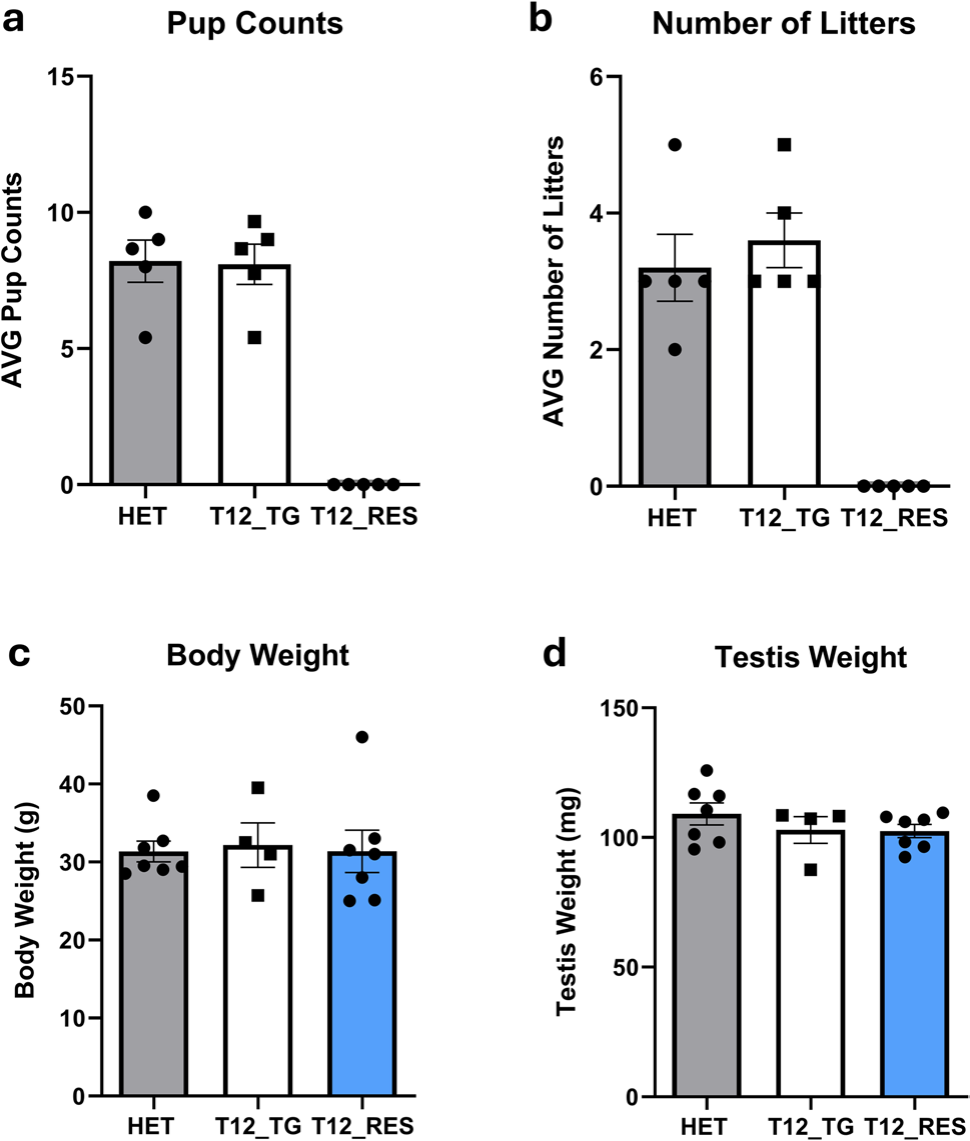
The *Tmprss12* transgene is not able to rescue fertility in *Tmprss12* KO mice. (**a**) Average litter per male per month, from natural mating of HET, T12 TG, and T12 RES mice. The average pup counts of T12 RES mice was 0, indicating it did not rescue fertility while the average pup counts between HET and T12 TG mice did not differ. (**b**) Average litter per male per month, from natural mating of HET, T12 TG, and T12 RES mice. Litter size was measured by the number of pups born. T12 RES mice had no litters and were unable to rescue fertility. (**c**) The average body weight from HET, T12 TG, and T12 RES mice. (**d**) The average testis weight from HET, T12 TG, and T12 RES mice.

To assess whether transgenic expression of human TMPRSS12 could rescue the infertility of *Tmprss12* KO mice, a 4-month fertility study was conducted. Sexually mature HET (*Tmprss12*^+*/d*^), TG [*Tmprss12*^+*/*+^*;Tg(CLGN*^*hTmprss12-3xFLAG*^*)*], and T12 RES [*Tmprss12*^*d/d*^*;Tg(CLGN*^*hTmprss12-3xFLAG*^*)*] male mice were continuously housed with *Tmprss12*^+*/d*^ or *Tmprss12*^*d/d*^ females, and litter production was monitored. HET males (N = 5) sired an average of 3.2 ± 0.49 litters per month, with an average of 8.21 ± 0.77 pups per litter (Fig. 6a,b). TG males (N = 5), which expressed both mouse *Tmprss12* and human *hTMPRSS12*, sired an average of 3.6 ± 0.40 litters per month, with an average of 8.09 ± 0.74 pups per litter, demonstrating no deleterious effect of the human transgene on fertility in the presence of endogenous mouse *Tmprss12* (Fig. 6a,b). In contrast, T12 RES males (N = 5) sired 0.00 ± 0.00 litters per month and 0.00 ± 0.00 pups per litter throughout the 4-month study period (Fig. 6a,b). No statistically significant differences were found in body weight (HET: 31.3 ± 1.3 g; TG: 32.2 ± 2.8 g; RES: 31.4 ± 2.7 g), testis weight (HET: 109.1 ± 4.2 mg; TG: 102.9 ± 5.1 mg; RES: 102.5 ± 2.5 mg), or epididymis weight (HET: 43.3 ± 1.5 mg; TG: 39.7 ± 2.4 mg; RES: 41.1 ± 2.4 mg) among the HET, TG, and T12 RES mouse lines (Fig. 6c,d).

## Discussion

The development of effective, non-hormonal contraceptives remains a significant goal to provide equitable options for family planning. Testis-specific proteins essential for male fertility represent promising targets for such contraceptives in males and sperm-specific proteins may be targets in the male and female reproductive tracks^8,12,16–23^. This study aimed to evaluate the potential of human *PRSS55* and human *TMPRSS12* to rescue the infertility phenotypes observed in their respective mouse knockout models, thereby establishing humanized mouse models for future contraceptive drug testing. Because these serine proteases are male reproductive tract specific^24^, we do not anticipate any on-target in vivo effects elsewhere with drug inhibitors. Our findings demonstrate that transgenic expression of human *PRSS55* can restore fertility in *Prss55* knockout mice, particularly when the protein is expressed with an intracellularly located tag. Conversely, the human *TMPRSS12* transgene failed to rescue infertility in *Tmprss12* knockout mice under the conditions tested.

Previous research has established PRSS55 as a serine protease critical for male fertility in mice, with its absence leading to sperm defects and an inability to traverse the UTJ. The present study confirms and extends these findings by demonstrating that human PRSS55 can functionally substitute for its mouse ortholog. Both humanized *PRSS55* transgenic lines, RES GPI (extracellular tag) and RES TM (intracellular tag), were capable of siring offspring in the *Prss55*^*d/d*^ background, which is otherwise infertile. Notably, the RES TM line, where the 3xFLAG tag was positioned intracellularly, exhibited a fertility profile comparable to the control TG_HET TM mice in terms of pups per litter, indicating a complete or near-complete rescue. In contrast, the RES GPI line, with an extracellularly positioned tag, showed a statistically significant reduction in the average number of pups per litter compared to its control, suggesting a partial rescue. This difference in rescue efficacy may be attributed to the location of the epitope tag. An extracellular tag, as in the RES GPI construct, might interfere with PRSS55’s proper localization on the sperm surface, its interaction with other essential proteins, or the processing of its substrates, which are critical for sperm function in the female reproductive tract. The more effective rescue by the RES TM construct suggests that minimizing potential steric hindrance to PRSS55’s extracellular domains is beneficial for its function.

Sperm parameter analysis (CASA) revealed that at 90 min post-extraction, both RES GPI and RES TM sperm displayed a significant decrease in hyperactivation compared to WT sperm. While hyperactivation is a critical step for fertilization, the overall successful fertility rescue, especially in the RES TM line, suggests that this observed difference in hyperactivation percentage in vitro does not preclude effective fertilization in vivo. Other key sperm parameters, such as count and progressive motility, were not significantly different, nor were body or testis weights, further supporting the viability of these humanized *PRSS55* lines. These results align with the critical role of PRSS55 in later stages of sperm function within the female tract, potentially related to ADAM3 processing or interaction with the zona pellucida, rather than initial motility patterns. Using the humanized *PRSS55* lines we looked at ADAM3 expression in both testis and epididymis tissue sections (Supplementary Fig. S5) and observed ADAM3 expression in the testis but did not see the same robust expression in the transgenic lines that we saw in WT mouse epididymis sections. From these findings we also wanted to confirm the presence of PRSS55 in *Adam3* KO mice (Supplementary Fig. S6), in these KO mice PRSS55 is present in both the testis and sperm. It is possible that the human protein in the mouse model might be bypassing ADAM3 processing or disrupting the ADAM3 cascade and that is why we are losing the robust expression in the transgenic mouse model.

TMPRSS12 is another testis-specific serine protease, and its absence in mice results in infertility^8^, characterized by impaired sperm motility and failure to migrate through the UTJ^6,7^. Our waveform analysis of sperm from *Tmprss12* KO mice corroborated these findings, revealing rigid and constrained flagellar movements compared to the robust, wide-arcing beats of WT sperm^25–27^. These observations are consistent with a severe motility defect. Despite confirmation of *hTMPRSS12* transgene mRNA expression in the testes of T12 RES mice, these mice remained infertile, siring no offspring during the 4-month mating trial. The CASA data from T12 RES males revealed significant impairments in multiple sperm parameters, including motility, hyperactivation, VAP, VCL, VSL, ALH, and BCF, at both 15 and 90 min post-extraction when compared to HET controls. These profound defects in sperm quality likely underlie the observed infertility.

Mouse and human TMPRSS12 precursors have a signal peptide, propeptide sequence, and a 271 amino acid mature sequence containing a 241 amino acid S1 peptidase domain (85% similar), 2 conserved N-linked oligosaccharides, and a transmembrane domain^28,29^. Mouse and human PRSS55 precursors have a signal peptide, a propeptide sequence, a ~ 330 amino acid mature sequence containing a ~ 233 amino acid S1 peptidase domain (68% similar), and a GPI anchor^30,31^. Although peptidase domains are very similar between human and mouse sequences the auxiliary motifs flanking the peptidase domain vary in sequence to a greater degree. This suggests additional roles/functions for those regions of the protein between mice and humans. Several factors may have contributed to the inability of human *TMPRSS12* to rescue the mouse knockout phenotype. Firstly, the gene sequence identity between human and mouse TMPRSS12 is relatively low, at approximately 60%^32^. This degree of divergence could lead to improper protein folding, instability, incorrect localization, or an inability to interact effectively with essential mouse partner proteins or substrates within the sperm or the female reproductive tract environment. While the presence of *hTMPRSS12* mRNA was confirmed, direct evidence of human TMPRSS12 protein expression and its quantity in the T12 RES mice sperm was not obtained in this study. This absence of protein expression data is a key limitation. Without confirmation that the human TMPRSS12 protein was present at appropriate levels and in the correct location, it cannot be definitively concluded that the human protein itself is non-functional in the mouse background; insufficient protein expression or mislocalization could also explain the lack of rescue. It is plausible that the human protein, even if expressed, could not integrate into the mouse molecular machinery required for sperm motility and function. The observation that TG mice (expressing both mouse *Tmprss12* and human *hTMPRSS12*) exhibited normal fertility indicates that the human transgene did not exert a dominant-negative effect. Future studies should incorporate methods to definitively detect and quantify the transgenic protein, for instance, by using validated antibodies against the human protein or the incorporated FLAG tag in sperm lysates, or by employing mass spectrometry.

For the *PRSS55* rescue lines, while fertility was rescued, the CASA results indicated differences in hyperactivation. The in vivo relevance of this specific in vitro finding could be explored further, although the successful production of litters suggests it is not a critical impediment to overall fertility. Additionally, while the *Clgn* promoter drives spermatid-specific expression, subtle differences in the timing or level of transgene expression compared to endogenous mouse gene expression cannot be entirely ruled out without more detailed quantitative analysis. The interpretation of the differential rescue success between the RES GPI and RES TM lines for PRSS55 is based on the presumed interference of the extracellular tag. While plausible, direct biochemical evidence of altered protein interactions or processing for the RES GPI construct was not pursued in this study.

This research successfully establishes that a humanized *PRSS55* transgene, particularly the RES TM construct, can effectively rescue the infertility phenotype in *Prss55* knockout mice. This achievement provides a valuable in vivo model system for testing potential non-hormonal male contraceptive compounds that target human PRSS55. The ability to test inhibitors against the human protein in a mouse physiological context is a crucial step in preclinical drug development. The failure of the human *TMPRSS12* transgene to rescue fertility, despite mRNA expression, underscores the challenges that can arise from interespecies differences in protein orthologs, particularly when complex protein–protein interactions are essential for function. Future attempts to create a humanized *TMPRSS12* rescue model might explore codon optimization for mouse expression systems, use of alternative expression constructs, or strategies to enhance protein stability or ensure correct processing and localization. Crucially, robust methods to confirm and quantify human protein expression in the target cells (sperm) will be essential for interpreting the outcomes of such experiments. Despite the negative rescue result for *TMPRSS12* in this specific humanized model, *TMPRSS12* remains a potentially viable target for male contraception due to its testis-specific expression and essential role in fertility. Further research might focus on developing inhibitors directly against mouse TMPRSS12 to validate its druggability in a homologous system before re-attempting humanized models or exploring alternative strategies for cross-species validation.

## Conclusion

In conclusion, this study demonstrates that human *PRSS55*, when expressed as a transgene with an intracellular tag, can functionally replace its mouse counterpart and restore fertility in *Prss55* knockout mice, providing a promising platform for human-specific contraceptive development. In contrast, the human *TMPRSS12* transgene did not rescue fertility in *Tmprss12* knockout mice, highlighting potential complexities in interspecies protein substitution, which may be compounded by unconfirmed protein expression levels in the relevant cells. These findings underscore the importance of *PRSS55* as a strong candidate for non-hormonal male contraception and suggest that further work is needed to develop a suitable humanized mouse model for TMPRSS12-targeted contraceptive research or to confirm adequate protein expression in future iterations of such models.

## Electronic supplementary material

Below is the link to the electronic supplementary material.

Supplementary Material 1

## Data Availability

Data is provided within the manuscript or supplementary information files.

## References

[1] Anderson, D. J. & Johnston, D. S. A brief history and future prospects of contraception. *Science* **380**, 154–158. 10.1126/science.adf9341 (2023). 37053322 10.1126/science.adf9341 PMC10615352

[2] Teal, S. & Edelman, A. Contraception selection, effectiveness, and adverse effects: A review. *JAMA* **326**, 2507–2518. 10.1001/jama.2021.21392 (2021). 34962522 10.1001/jama.2021.21392

[3] Thirumalai, A. & Page, S. T. Male hormonal contraception. *Annu. Rev. Med.* **71**, 17–31. 10.1146/annurev-med-042418-010947 (2020). 31537185 10.1146/annurev-med-042418-010947

[4] Kobayashi, K. et al. Prss55 but not Prss51 is required for male fertility in micedagger. *Biol. Reprod.* **103**, 223–234. 10.1093/biolre/ioaa041 (2020). 32301961 10.1093/biolre/ioaa041 PMC7401375

[5] Sutton, C. et al. Molecular dissection and testing of PRSS37 function through LC-MS/MS and the generation of a PRSS37 humanized mouse model. *Sci. Rep.* **13**, 11374. 10.1038/s41598-023-37700-1 (2023). 37452050 10.1038/s41598-023-37700-1 PMC10349139

[6] Larasati, T. et al. Tmprss12 is required for sperm motility and uterotubal junction migration in micedagger. *Biol. Reprod.* **103**, 254–263. 10.1093/biolre/ioaa060 (2020). 32529245 10.1093/biolre/ioaa060 PMC7401031

[7] Zhang, J. et al. TMPRSS12 functions in meiosis and spermiogenesis and is required for male fertility in mice. *Front. Cell Dev. Biol.*10.3389/fcell.2022.757042 (2022). 35547804 10.3389/fcell.2022.757042 PMC9081376

[8] Kobayashi, K. et al. Prss55 but not Prss51 is required for male fertility in mice†. *Biol. Reprod.* **103**, 223–234. 10.1093/biolre/ioaa041 (2020). 32301961 10.1093/biolre/ioaa041 PMC7401375

[9] Zhu, F. et al. PRSS55 plays an important role in the structural differentiation and energy metabolism of sperm and is required for male fertility in mice. *J. Cell Mol. Med.* **25**, 2040–2051. 10.1111/jcmm.16116 (2021). 33417308 10.1111/jcmm.16116 PMC7882947

[10] Li, C. et al. Spem2, a novel testis-enriched gene, is required for spermiogenesis and fertilization in mice. *Cell. Mol. Life Sci.*10.1007/s00018-024-05147-w (2024). 38421455 10.1007/s00018-024-05147-w PMC10904452

[11] Percie du Sert, N. et al. The ARRIVE guidelines 2.0: Updated guidelines for reporting animal research. *PLOS Biol.* **18**, e3000410. 10.1371/journal.pbio.3000410 (2020). 32663219 10.1371/journal.pbio.3000410 PMC7360023

[12] Ikawa, M. et al. Calsperin is a testis-specific chaperone required for sperm fertility. *J. Biol. Chem.* **286**, 5639–5646. 10.1074/jbc.M110.140152 (2011). 21131354 10.1074/jbc.M110.140152 PMC3037677

[13] Oura, S., Ninomiya, A., Sugihara, F., Matzuk, M. M. & Ikawa, M. Proximity-dependent biotin labeling in testicular germ cells identified TESMIN-associated proteins. *Sci. Rep.* **12**, 22198. 10.1038/s41598-022-26501-7 (2022). 36564444 10.1038/s41598-022-26501-7 PMC9789103

[14] Kim, B. J. et al. Genome-wide reinforcement of cohesin binding at pre-existing cohesin sites in response to ionizing radiation in human cells. *J. Biol. Chem.* **285**, 22784–22792. 10.1074/jbc.M110.134577 (2010). 20501661 10.1074/jbc.M110.134577 PMC2906269

[15] Nozawa, K. et al. The testis-specific E3 ubiquitin ligase RNF133 is required for fecundity in mice. *BMC Biol.*10.1186/s12915-022-01368-2 (2022). 35831855 10.1186/s12915-022-01368-2 PMC9277888

[16] Robertson, M. J. et al. Large-scale discovery of male reproductive tract-specific genes through analysis of RNA-seq datasets. *BMC Biol.* **18**, 103. 10.1186/s12915-020-00826-z (2020). 32814578 10.1186/s12915-020-00826-z PMC7436996

[17] Shen, C. et al. Prss37 is required for male fertility in the mouse. *Biol. Reprod.* **88**, 123. 10.1095/biolreprod.112.107086 (2013). 23553430 10.1095/biolreprod.112.107086

[18] Xiong, W. et al. Dissecting the PRSS37 interactome and potential mechanisms leading to ADAM3 loss in PRSS37-null sperm. *J. Cell Sci.* **134**, jcs258426. 10.1242/jcs.258426 (2021). 34028541 10.1242/jcs.258426

[19] Xiong, W., Wang, Z. & Shen, C. An update of the regulatory factors of sperm migration from the uterus into the oviduct by genetically manipulated mice. *Mol. Reprod. Dev.* **86**, 935–955. 10.1002/mrd.23180 (2019). 31131960 10.1002/mrd.23180

[20] Vandenbrouck, Y. et al. Looking for missing proteins in the proteome of human spermatozoa: An update. *J. Proteome Res.* **15**, 3998–4019. 10.1021/acs.jproteome.6b00400 (2016). 27444420 10.1021/acs.jproteome.6b00400

[21] Yamaguchi, R. et al. Disruption of ADAM3 impairs the migration of sperm into oviduct in mouse1. *Biol. Reprod.* **81**, 142–146. 10.1095/biolreprod.108.074021 (2009). 19339711 10.1095/biolreprod.108.074021

[22] Yamaguchi, R., Yamagata, K., Ikawa, M., Moss, S. B. & Okabe, M. Aberrant distribution of ADAM3 in sperm from both angiotensin-converting enzyme (Ace)- and calmegin (Clgn)-deficient mice1. *Biol. Reprod.* **75**, 760–766. 10.1095/biolreprod.106.052977 (2006). 16870943 10.1095/biolreprod.106.052977

[23] Tokuhiro, K., Ikawa, M., Benham, A. M. & Okabe, M. Protein disulfide isomerase homolog PDILT is required for quality control of sperm membrane protein ADAM3 and male fertility [corrected]. *Proc. Natl. Acad. Sci. U.S.A.* **109**, 3850–3855. 10.1073/pnas.1117963109 (2012). 22357757 10.1073/pnas.1117963109 PMC3309714

[24] Robertson, M. J. et al. Large-scale discovery of male reproductive tract-specific genes through analysis of RNA-seq datasets. *BMC Biol.*10.1186/s12915-020-00826-z (2020). 32814578 10.1186/s12915-020-00826-z PMC7436996

[25] Gallagher, M. T., Cupples, G., Ooi, E. H., Kirkman-Brown, J. C. & Smith, D. J. Rapid sperm capture: high-throughput flagellar waveform analysis. 10.1093/humrep/dez056 PMC6613345 31170729

[26] Vyklicka, L. & Lishko, P. V. Dissecting the signaling pathways involved in the function of sperm flagellum. 10.1016/j.ceb.2020.01.015 PMC7296462 32097833

[27] Tufoni, C. et al. Flagellar beating forces of human spermatozoa with different motility behaviors. 10.1186/s12958-024-01197-8 PMC10916019 38448984

[28] *TMPSC_Human*, https://www.uniprot.org/uniprotkb/Q86WS5/entry

[29] *TMPSC_Mouse*, https://www.uniprot.org/uniprotkb/Q3V0Q7/entry

[30] *PRSS55_Human*, https://www.uniprot.org/uniprotkb/Q14BX2/entry#sequences

[31] *PRSS55_Mouse* https://www.uniprot.org/uniprotkb/Q14BX2/entry

[32] e!Ensembl. *Gene: TMPRSS12 ENSMUSG00000045631*, https://useast.ensembl.org/Mus_musculus/Gene/Compara_Ortholog/Alignment?db=core;g=ENSMUSG00000045631;g1=ENSG00000186452;hom_id=90452738;r=15:100178718-100190947 (2024).

