# Supplementary material for: Rescue of male infertility by human PRSS55 in transgenic mice establishes a contraceptive research model
Source: Sci Rep. 2025 Aug 5;15:28657. doi: 10.1038/s41598-025-09604-9 (PMC12325665; doi:10.1038/s41598-025-09604-9)
Supplement: Supplementary file 1 — Supplementary Material 1 [file 41598_2025_9604_MOESM1_ESM.pdf]

Supplementary Figures

Rescue of male infertility by human PRSS55 in transgenic mice establishes a contraceptive research model

Fig S1. CASA results from RES GPI mice

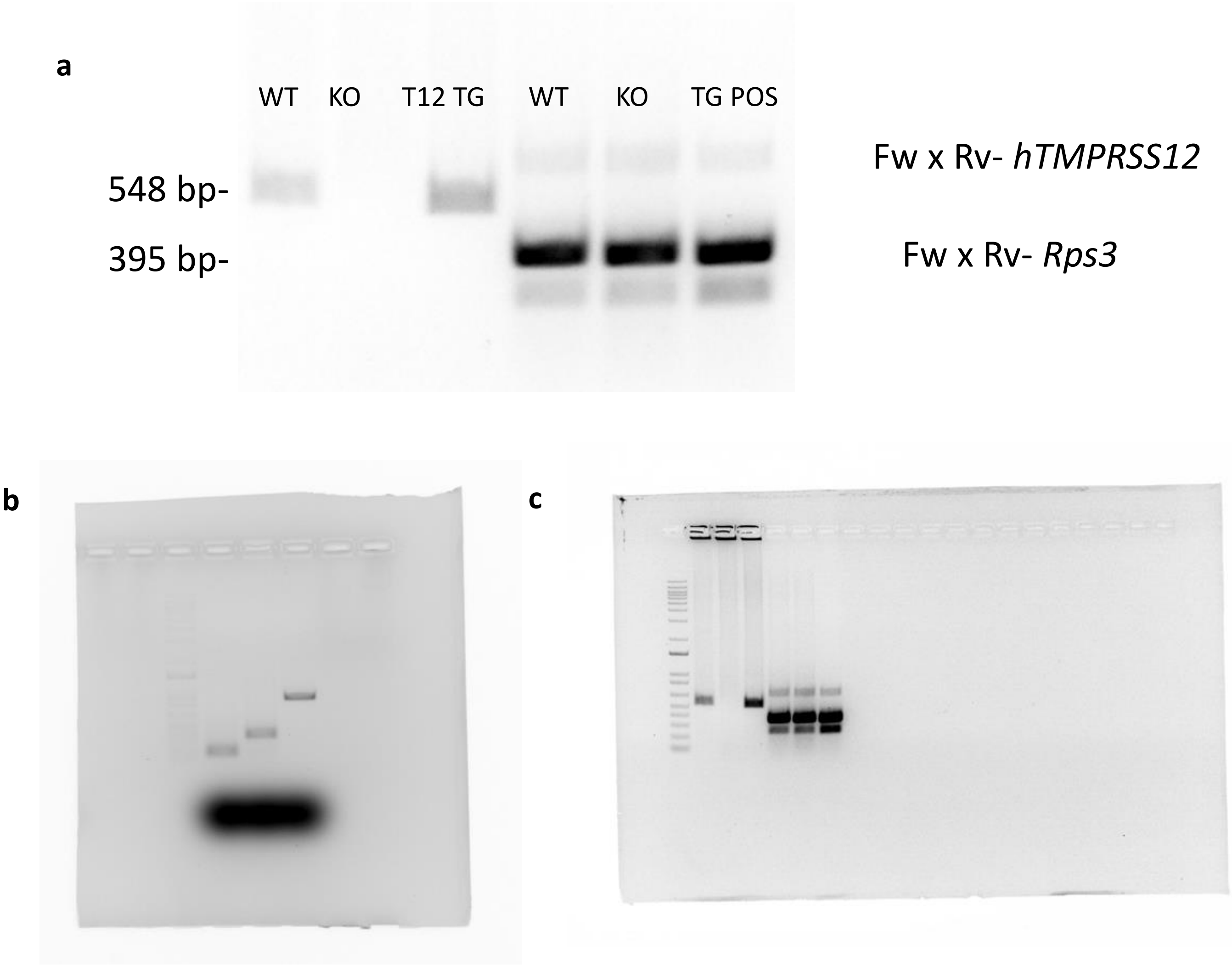

Figure S1. TMPRSS12 confirmation of the transgene and uncut blots

a. Gel showing the results of extracting RNA from the testis of WT, KO, and RES testes after producing cDNA and running PCR with *hTMPRSS12* (lanes 1-3) and *Rsp3* (lanes 4-6), the full gel can be found in Supplementary Fig. S1.

b. Gel image that shows genotyping results from a T12 WT, T12 KO, and T12 TG, respectively.

c. Gel image that shows results from collecting cDNA form testis from a WT, KO, and RES with lanes 2-4 showing results of hTmprss12 and lanes 5-7 showing the control Rps3.

Fig S2. CASA results from RES GPI mice

a CASA at 15 minutes post dissection

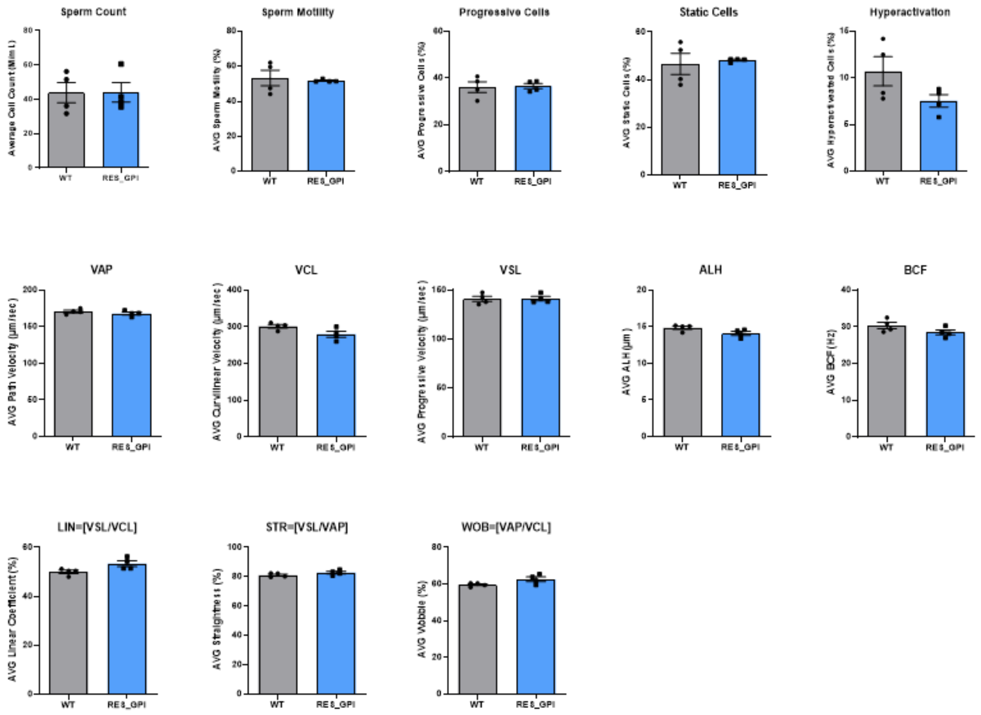

b CASA at 90 minutes post dissection

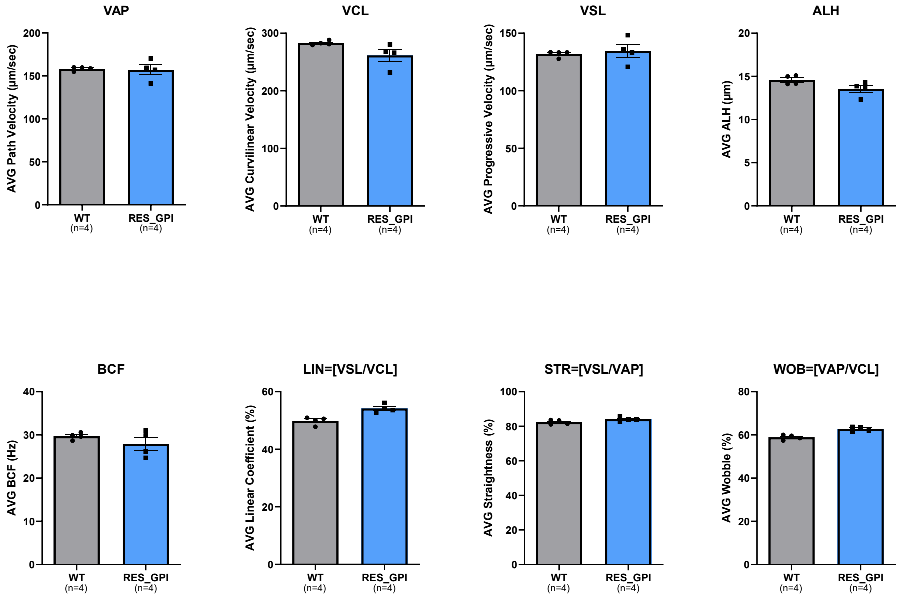

Figure S2. CASA results from RES GPI mice

a. CASA parameters between RES GPI mice at 15 minutes post dissection.

b. CASA parameters between RES GPI mice at 90 minutes post dissection.

Fig S3. CASA results from RES TM mice

a CASA at 15 minutes post dissection

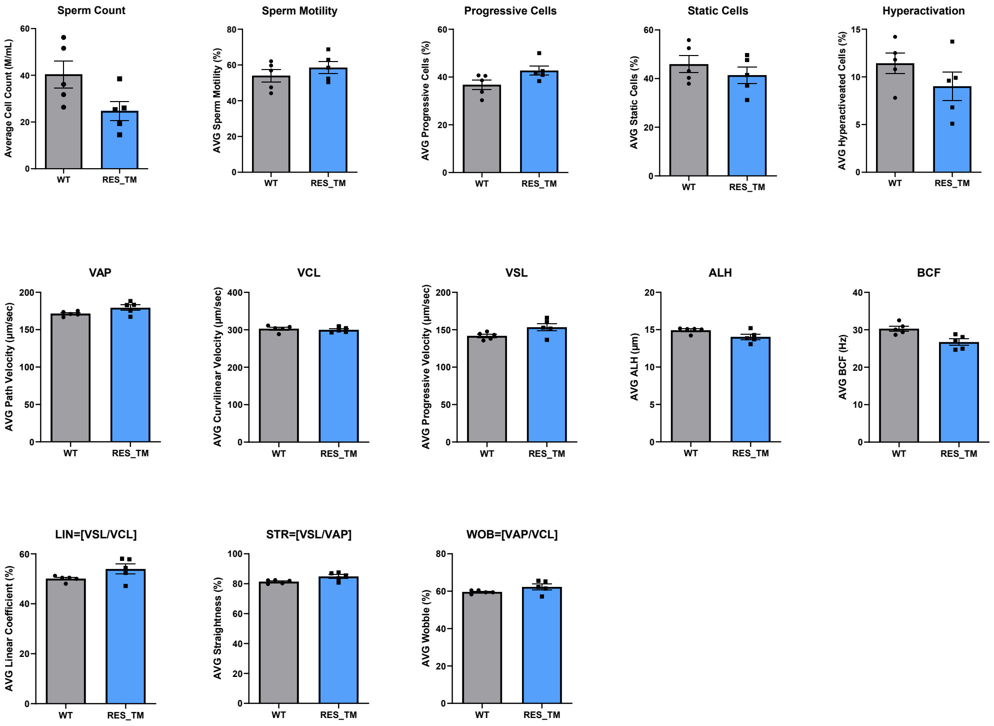

b CASA at 90 minutes post dissection

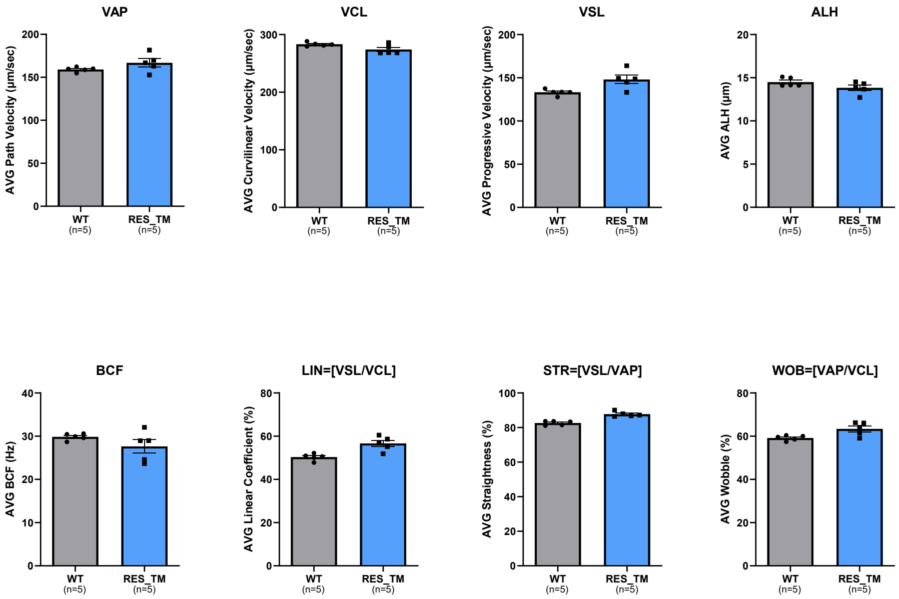

Figure S3. CASA results from RES TM mice

a. CASA parameters between RES TM mice at 15 minutes post dissection.

b. CASA parameters between RES TM mice at 90 minutes post dissection.

Fig S4. Uncut/cropped gels and blots

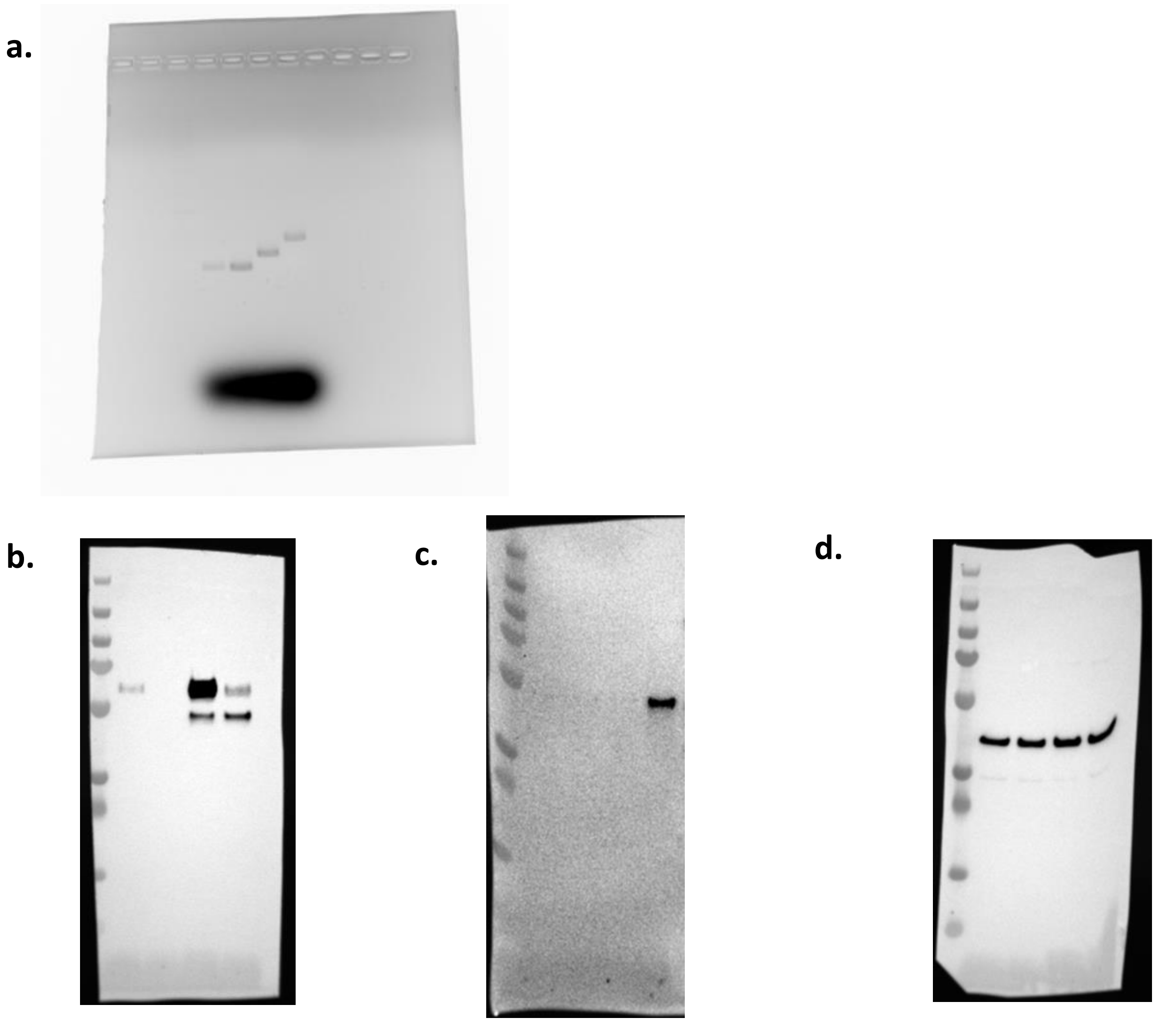

Figure S4. Uncut/cropped gels and blots

a. Gel of *Prss55* genotyping; WT, KO, RES GPI, and RES TM , respectively.

b. Blot of Anti-PRSS55 WT, KO, RES GPI, and RES TM in lanes 1-4, respectively.

c. Blot of Anti-FLAG WT, KO, RES GPI, and RES TM in lanes 1-4, respectively.

d. Blot of Anti-GAPDH WT, KO, RES GPI, and RES TM in lanes 1-4, respectively.

Fig S5. ADAM3 expression in *PRSS55* transgenic mouse lines

a.

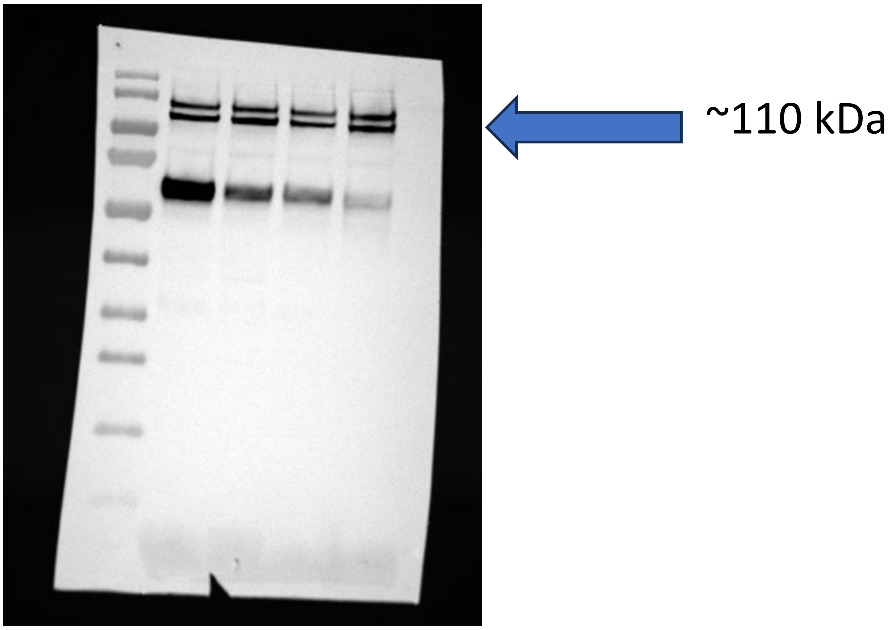

b.

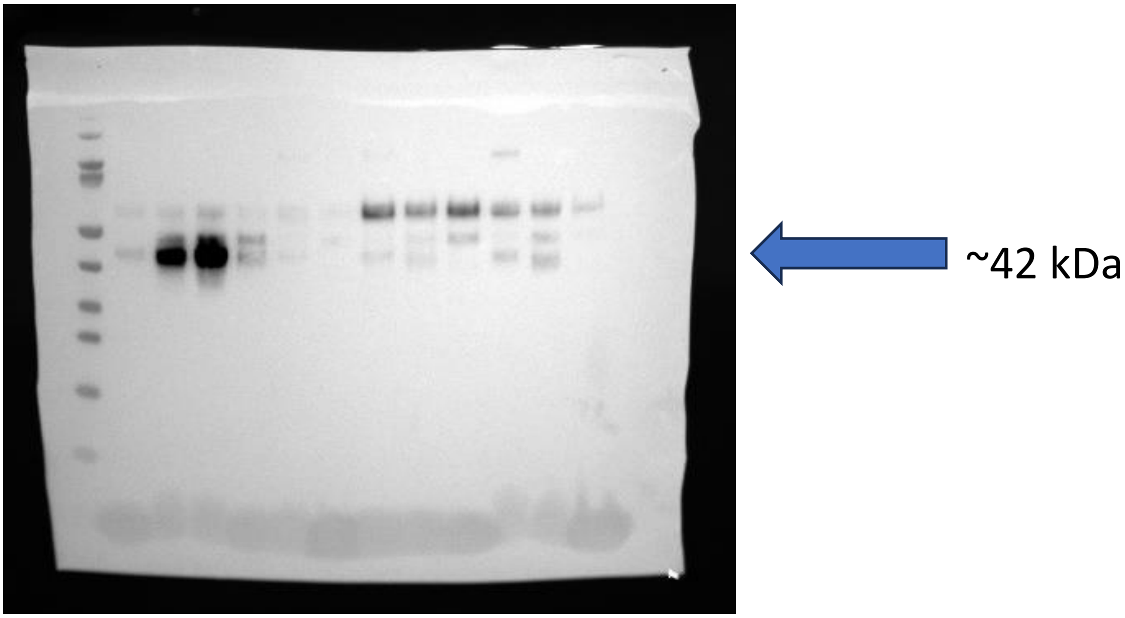

Figure S5. ADAM3 expression in *PRSS55* transgenic mouse lines

a. Blot of Anti-ADAM3 in testis samples from WT, KO, RES GPI, and RES TM in lanes 1-4, respectively.

b. Blot of Anti-ADAM3 in epididymis tissue samples, lane 1 ladder, lanes 2-4 WT, lanes 5-7 PRSS55 KO, lanes 8-10 RES GPI, lanes 11-13 RES TM (caput, corpus, cauda; respectively)

Fig S6. PRSS55 is present in the testis and sperm of *Adam3* knockout mice.

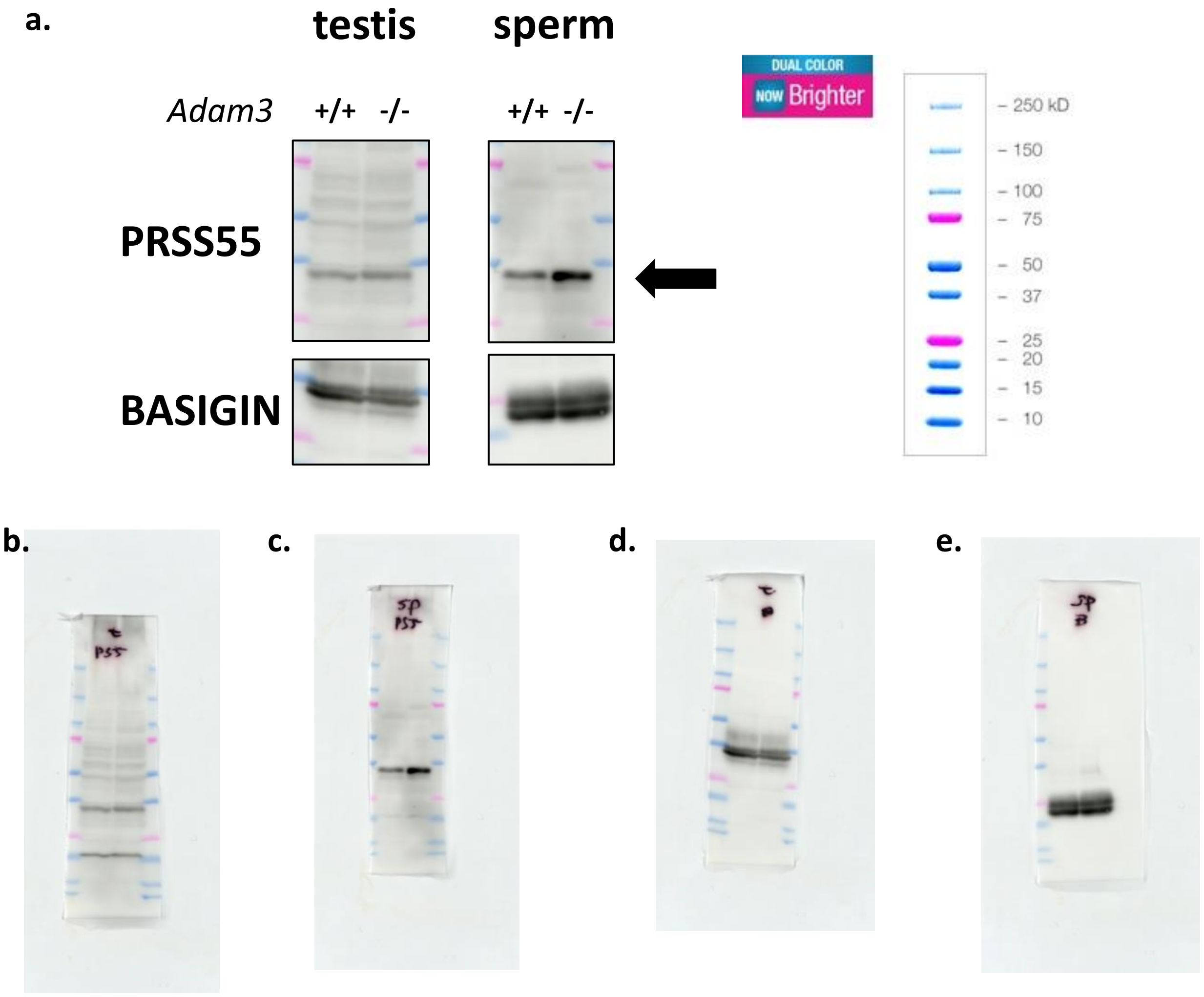

Figure S6. PRSS55 is present in the testis and sperm of *Adam3* knockout mice

a. Blot of Anti-PRSS55 in testis samples from WT, KO mice in the testis and sperm.

b. Uncut blot of Anti-PRSS55 on testis tissue in WT and KO *Adam3* mice

c. Uncut blot of Anti-PRSS55 on sperm cells from WT and KO *Adam3* mice

d. Uncut blot of Anti-BASIGIN on testis tissue from WT and KO *Adam3* mice

e. Uncut blot of Anti-BASIGIN on sperm cells from WT and KO *Adam3* mice

Fig S7. Identification of TMPRSS12 through immunoblot was not successful.

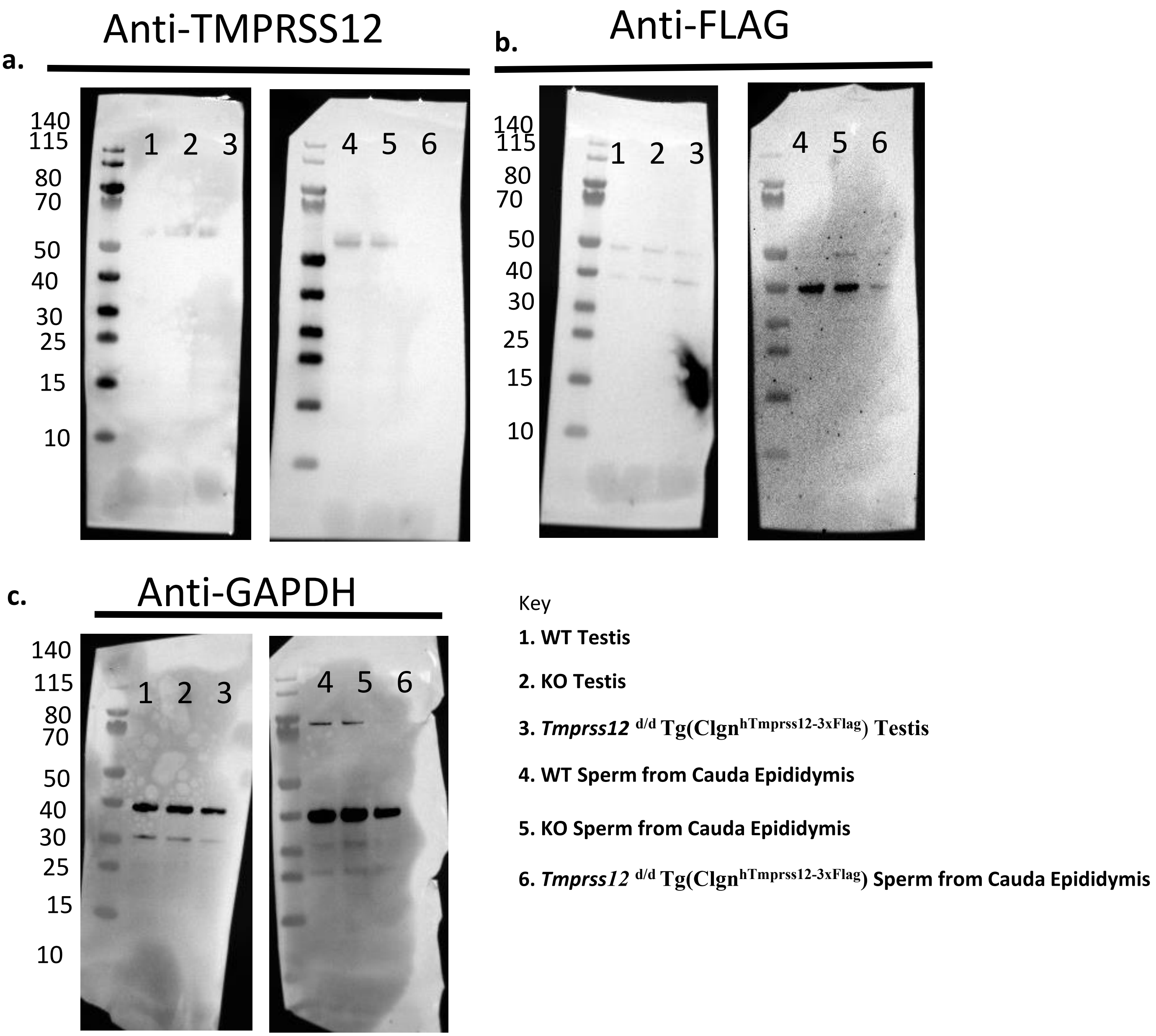

Figure S7. Uncut/cropped gels and blots

a. Blot of Anti-TMPRSS12 WT, KO, RES T12..

b. Blot of Anti-FLAG WT, KO, RES T12

c. Blot of Anti-GAPDH WT, KO, RES T12

Fig S8. Transgenic mouse constructs in pCAG vector.

**a.**	pCAG_PRSS55_3XFLAG_GPI	GACATTGATTATTGACTAGTTATTAATAGTAATCAATTACGGGGTCATTAGTTCATAGCCCATATATGGAGTTCCGCGTTACATAACTTACGGTAAATGGCCCGCCTGGCTGACCGC<br>CCAACGACCCCCGCCCATTGACGTCAATAATGACGTATGTTCCCATAGTAACGCCAATAGGGACTTTCCATTGACGTCAATGGGTGGACTATTTACGGTAAACTGCCCACTTGGCAG<br>TACATCAAGTGTATCATATGCCAAGTACGCCCCCTATTGACGTCAATGACGGTAAATGGCCCGCCTGGCATTATGCCCAGTACATGACCTTATGGGACTTTCCTACTTGGCAGTACA<br>TCTACGTATTAGTCATCGCTATTACCATGGTCGAGGTGAGCCCCACGTTCTGCTTCACTCTCCCCATCTCCCCCCCCTCCCCACCCCCAATTTTGTATTTATTTATTTTTTAATTA<br>TTTTGTGCAGCGATGGGGGCGGGGGGGGGGGGGGCGCGCGCCAGGCGGGGCGGGGCGGGGCGAGGGGCGGGGCGGGGCGAGGCGGAGAGGTGCGGCGGCAGCCAATCAGAGCGGCGC<br>GCTCCGAAAGTTTCCTTTTATGGCGAGGCGGCGGCGGCGGCGGCCCTATAAAAAGCGAAGCGCGCGGCGGGCGGGAGTCGCTGCGTTGCCTTCGCCCCGTGCCCCGCTCCGCGCCGC<br>CTCGCGCCGCCCGCCCCGGCTCTGACTGACCGCGTTACTCCCACAGGTGAGCGGGCGGGACGGCCCTTCTCCTCCGGGCTGTAATTAGCGCTTGGTTTAATGACGGCTCGTTTCTTT<br>TCTGTGGCTGCGTGAAAGCCTTAAAGGGCTCCGGGAGGGCCCTTTGTGCGGGGGGGAGCGGCTCGGGGGGTGCGTGCGTGTGTGTGTGCGTGGGGAGCGCCGCGTGCGGCCCGCGCT<br>GCCCGGCGGCTGTGAGCGCTGCGGGCGCGGCGCGGGGCTTTGTGCGCTCCGCGTGTGCGCGAGGGGAGCGCGGCCGGGGGCGGTGCCCCGCGGTGCGGGGGGGCTGCGAGGGGAACA<br>AAGGCTGCGTGCGGGGTGTGTGCGTGGGGGGGTGAGCAGGGGGTGTGGGCGCGGCGGTCGGGCTGTAACCCCCCCCTGCACCCCCCTCCCCGAGTTGCTGAGCACGGCCCGGCTTCG<br>GGTGCGGGGCTCCGTGCGGGGCGTGGCGCGGGGCTCGCCGTGCCGGGCGGGGGGTGGCGGCAGGTGGGGGTGCCGGGCGGGGCGGGGCCGCCTCGGGCCGGGGAGGGCTCGGGGGAG<br>GGGCGCGGCGGCCCCGGAGCGCCGGCGGCTGTCGAGGCGCGGCGAGCCGCAGCCATTGCCTTTTATGGTAATCGTGCGAGAGGGCGCAGGGACTTCCTTTGTCCCAAATCTGGCGGA<br>GCCGAAATCTGGGAGGCGCCGCCGCACCCCCTCTAGCGGGCGCGGGCGAAGCGGTGCGGCGCCGGCAGGAAGGAAATGGGCGGGGAGGGCCTTCGTGCGTCGCCGCGCCGCCGTCCC<br>CTTCTCCATCTCCAGCCTCGGGGCTGCCGCAGGGGGACGGCTGCCTTCGGGGGGGACGGGGCAGGGCGGGGTTCGGCTTCTGGCGTGTGACCGGCGGCTCTAGCCTCTGCTAACCAT<br>GTTCATGCCTTCTTCTTTTTCCTACAGCTCCTGGGCAACGTGCTGGTTGTTGTGCTGTCTCATCATTTTGGCAAATCTAGAGCCGCCACCATGCTCCTGTTCTCAGTGTTGCTGCTC<br>CTGTCCCTGGTCACGGGAACTCAGCTCGGTCCACGGACTCCTCTCCCAGAGGCTGGAGTGGCTATCCTAGGCAGGGCTAGGGGAGCCCACCGCCCTCAGCCCCCTCATCCCCCCAGC<br>CCAGTCAGTGAATGTGGTGACAGATCTATTTTCGAGGGAAGAACTCGGTATTCCAGAATCACAGGGGGGATGGAGGCGGAGGTGGGTGAGTTTCCGTGGCAGGTGAGTATTCAGGCA<br>AGAAGTGAACCTTTCTGTGGCGGCTCCATCCTCAACAAGTGGTGGATTCTCACTGCGGCTCACTGCTTATATTCCGAGGAGCTGTTTCCAGAAGAACTGAGTGTCGTGCTGGGGACC<br>AACGACTTAACTAGCCCATCCATGGAAATAAAGGAGGTCGCCAGCATCATTCTTCACAAAGACTTTAAGAGAGCCAACATGGACAATGACATTGCCTTGCTGCTGCTGGCTTCGCCC<br>ATCAAGCTCGATGACCTGAAGGTGCCCATCTGCCTCCCCACGCAGCCCGGCCCTGCCACATGGCGCGAATGCTGGGTGGCAGGTTGGGGCCAGACCAATGCTGCTGACAAAAACTCT<br>GTGAAAACGGATCTGATGAAAGCGCCAATGGTCATCATGGACTGGGAGGAGTGTTCAAAGATGTTTCCAAAACTTACCAAAAATATGCTGTGTGCCGGATACAAGAATGAGAGCTAT<br>GATGCCTGCAAGGGTGACAGTGGGGGGCCTCTGGTCTGCACCCCAGAGCCTGGTGAGAAGTGGTACCAGGTGGGCATCATAAGCTGGGGAAAGAGCTGTGGAGAGAAGAACACCCCA<br>GGGATATACACCTCGTTGGTGAACTACAACCTCTGGATCGAGAAAGTGACCCAGCTAGAGGGCAGGCCCTTCAATGCAGAGAAAAGGAGGACTTCTGTCAAACAGAAACCTATGGGC<br>GGCGGAGGCGGGTCCGACTACAAAGACCATGACGGTGATTATAAAGATCATGACATTGATTACAAGGATGACGATGACAAGGGCGGAGGCGGGTCCTCCCCAGTCTCGGGAGTCCCA<br>GAGCCAGGCAGCCCCAGATCCTGGCTCCTGCTCTGTCCCCTGTCCCATGTGTTGTTCAGAGCTATTTTGTACTGA
**b.**	pCAG_PRSS55_TM_3XFLAG	GACATTGATTATTGACTAGTTATTAATAGTAATCAATTACGGGGTCATTAGTTCATAGCCCATATATGGAGTTCCGCGTTACATAACTTACGGTAAATGGCCCGCCTGGCTGACCGC<br>CCAACGACCCCCGCCCATTGACGTCAATAATGACGTATGTTCCCATAGTAACGCCAATAGGGACTTTCCATTGACGTCAATGGGTGGACTATTTACGGTAAACTGCCCACTTGGCAG<br>TACATCAAGTGTATCATATGCCAAGTACGCCCCCTATTGACGTCAATGACGGTAAATGGCCCGCCTGGCATTATGCCCAGTACATGACCTTATGGGACTTTCCTACTTGGCAGTACA<br>TCTACGTATTAGTCATCGCTATTACCATGGTCGAGGTGAGCCCCACGTTCTGCTTCACTCTCCCCATCTCCCCCCCCTCCCCACCCCCAATTTTGTATTTATTTATTTTTTAATTA<br>TTTTGTGCAGCGATGGGGGCGGGGGGGGGGGGGGCGCGCGCCAGGCGGGGCGGGGCGGGGCGAGGGGCGGGGCGGGGCGAGGCGGAGAGGTGCGGCGGCAGCCAATCAGAGCGGCGC<br>GCTCCGAAAGTTTCCTTTTATGGCGAGGCGGCGGCGGCGGCGGCCCTATAAAAAGCGAAGCGCGCGGCGGGCGGGAGTCGCTGCGTTGCCTTCGCCCCGTGCCCCGCTCCGCGCCGC<br>CTCGCGCCGCCCGCCCCGGCTCTGACTGACCGCGTTACTCCCACAGGTGAGCGGGCGGGACGGCCCTTCTCCTCCGGGCTGTAATTAGCGCTTGGTTTAATGACGGCTCGTTTCTTT<br>TCTGTGGCTGCGTGAAAGCCTTAAAGGGCTCCGGGAGGGCCCTTTGTGCGGGGGGGAGCGGCTCGGGGGGTGCGTGCGTGTGTGTGTGCGTGGGGAGCGCCGCGTGCGGCCCGCGCT<br>GCCCGGCGGCTGTGAGCGCTGCGGGCGCGGCGCGGGGCTTTGTGCGCTCCGCGTGTGCGCGAGGGGAGCGCGGCCGGGGGCGGTGCCCCGCGGTGCGGGGGGGCTGCGAGGGGAACA<br>AAGGCTGCGTGCGGGGTGTGTGCGTGGGGGGGTGAGCAGGGGGTGTGGGCGCGGCGGTCGGGCTGTAACCCCCCCCTGCACCCCCCTCCCCGAGTTGCTGAGCACGGCCCGGCTTCG<br>GGTGCGGGGCTCCGTGCGGGGCGTGGCGCGGGGCTCGCCGTGCCGGGCGGGGGGTGGCGGCAGGTGGGGGTGCCGGGCGGGGCGGGGCCGCCTCGGGCCGGGGAGGGCTCGGGGGAG<br>GGGCGCGGCGGCCCCGGAGCGCCGGCGGCTGTCGAGGCGCGGCGAGCCGCAGCCATTGCCTTTTATGGTAATCGTGCGAGAGGGCGCAGGGACTTCCTTTGTCCCAAATCTGGCGGA<br>GCCGAAATCTGGGAGGCGCCGCCGCACCCCCTCTAGCGGGCGCGGGCGAAGCGGTGCGGCGCCGGCAGGAAGGAAATGGGCGGGGAGGGCCTTCGTGCGTCGCCGCGCCGCCGTCCC<br>CTTCTCCATCTCCAGCCTCGGGGCTGCCGCAGGGGGACGGCTGCCTTCGGGGGGGACGGGGCAGGGCGGGGTTCGGCTTCTGGCGTGTGACCGGCGGCTCTAGCCTCTGCTAACCAT<br>GTTCATGCCTTCTTCTTTTTCCTACAGCTCCTGGGCAACGTGCTGGTTGTTGTGCTGTCTCATCATTTTGGCAAATCTAGAGCCGCCACCATGCTCCTGTTCTCAGTGTTGCTGCTC<br>CTGTCCCTGGTCACGGGAACTCAGCTCGGTCCACGGACTCCTCTCCCAGAGGCTGGAGTGGCTATCCTAGGCAGGGCTAGGGGAGCCCACCGCCCTCAGCCCCCTCATCCCCCCAGC<br>CCAGTCAGTGAATGTGGTGACAGATCTATTTTCGAGGGAAGAACTCGGTATTCCAGAATCACAGGGGGGATGGAGGCGGAGGTGGGTGAGTTTCCGTGGCAGGTGAGTATTCAGGCA<br>AGAAGTGAACCTTTCTGTGGCGGCTCCATCCTCAACAAGTGGTGGATTCTCACTGCGGCTCACTGCTTATATTCCGAGGAGCTGTTTCCAGAAGAACTGAGTGTCGTGCTGGGGACC<br>AACGACTTAACTAGCCCATCCATGGAAATAAAGGAGGTCGCCAGCATCATTCTTCACAAAGACTTTAAGAGAGCCAACATGGACAATGACATTGCCTTGCTGCTGCTGGCTTCGCCC<br>ATCAAGCTCGATGACCTGAAGGTGCCCATCTGCCTCCCCACGCAGCCCGGCCCTGCCACATGGCGCGAATGCTGGGTGGCAGGTTGGGGCCAGACCAATGCTGCTGACAAAAACTCT<br>GTGAAAACGGATCTGATGAAAGCGCCAATGGTCATCATGGACTGGGAGGAGTGTTCAAAGATGTTTCCAAAACTTACCAAAAATATGCTGTGTGCCGGATACAAGAATGAGAGCTAT<br>GATGCCTGCAAGGGTGACAGTGGGGGGCCTCTGGTCTGCACCCCAGAGCCTGGTGAGAAGTGGTACCAGGTGGGCATCATAAGCTGGGGAAAGAGCTGTGGAGAGAAGAACACCCCA<br>GGGATATACACCTCGTTGGTGAACTACAACCTCTGGATCGAGAAAGTGACCCAGCTAGAGGGCAGGCCCTTCAATGCAGAGAAAAGGAGGACTTCTGTCAAACAGAAACCTATGGGC<br>TCCCCAGTCATACTTACTATAAATATTTTACGTGGCCAGATCCTCATAGCTTTATGTTTTGTCATCTTACTAGCAACAACAGGCGGAGGCGGGTCCGGAGGCGGGGGCAGCGGCGGG<br>GGCGGAAGCGGGGGCGGAGGCTCCGGAGGCGGAGGGAGCGACTACAAAGACCATGACGGTGATTATAAAGATCATGACATTGATTACAAGGATGACGATGACAAGTGA
**c.**	pCAG_TMPRSS12_3XFLAG	GACATTGATTATTGACTAGTTATTAATAGTAATCAATTACGGGGTCATTAGTTCATAGCCCATATATGGAGTTCCGCGTTACATAACTTACGGTAAATGGCCCGCCTGGCTGACCGC<br>CCAACGACCCCCGCCCATTGACGTCAATAATGACGTATGTTCCCATAGTAACGCCAATAGGGACTTTCCATTGACGTCAATGGGTGGACTATTTACGGTAAACTGCCCACTTGGCAG<br>TACATCAAGTGTATCATATGCCAAGTACGCCCCCTATTGACGTCAATGACGGTAAATGGCCCGCCTGGCATTATGCCCAGTACATGACCTTATGGGACTTTCCTACTTGGCAGTACA<br>TCTACGTATTAGTCATCGCTATTACCATGGTCGAGGTGAGCCCCACGTTCTGCTTCACTCTCCCCATCTCCCCCCCCTCCCCACCCCCAATTTTGTATTTATTTATTTTTTAATTA<br>TTTTGTGCAGCGATGGGGGCGGGGGGGGGGGGGGCGCGCGCCAGGCGGGGCGGGGCGGGGCGAGGGGCGGGGCGGGGCGAGGCGGAGAGGTGCGGCGGCAGCCAATCAGAGCGGCGC<br>GCTCCGAAAGTTTCCTTTTATGGCGAGGCGGCGGCGGCGGCGGCCCTATAAAAAGCGAAGCGCGCGGCGGGCGGGAGTCGCTGCGTTGCCTTCGCCCCGTGCCCCGCTCCGCGCCGC<br>CTCGCGCCGCCCGCCCCGGCTCTGACTGACCGCGTTACTCCCACAGGTGAGCGGGCGGGACGGCCCTTCTCCTCCGGGCTGTAATTAGCGCTTGGTTTAATGACGGCTCGTTTCTTT<br>TCTGTGGCTGCGTGAAAGCCTTAAAGGGCTCCGGGAGGGCCCTTTGTGCGGGGGGGAGCGGCTCGGGGGGTGCGTGCGTGTGTGTGTGCGTGGGGAGCGCCGCGTGCGGCCCGCGCT<br>GCCCGGCGGCTGTGAGCGCTGCGGGCGCGGCGCGGGGCTTTGTGCGCTCCGCGTGTGCGCGAGGGGAGCGCGGCCGGGGGCGGTGCCCCGCGGTGCGGGGGGGCTGCGAGGGGAACA<br>AAGGCTGCGTGCGGGGTGTGTGCGTGGGGGGGTGAGCAGGGGGTGTGGGCGCGGCGGTCGGGCTGTAACCCCCCCCTGCACCCCCCTCCCCGAGTTGCTGAGCACGGCCCGGCTTCG<br>GGTGCGGGGCTCCGTGCGGGGCGTGGCGCGGGGCTCGCCGTGCCGGGCGGGGGGTGGCGGCAGGTGGGGGTGCCGGGCGGGGCGGGGCCGCCTCGGGCCGGGGAGGGCTCGGGGGAG<br>GGGCGCGGCGGCCCCGGAGCGCCGGCGGCTGTCGAGGCGCGGCGAGCCGCAGCCATTGCCTTTTATGGTAATCGTGCGAGAGGGCGCAGGGACTTCCTTTGTCCCAAATCTGGCGGA<br>GCCGAAATCTGGGAGGCGCCGCCGCACCCCCTCTAGCGGGCGCGGGCGAAGCGGTGCGGCGCCGGCAGGAAGGAAATGGGCGGGGAGGGCCTTCGTGCGTCGCCGCGCCGCCGTCCC<br>CTTCTCCATCTCCAGCCTCGGGGCTGCCGCAGGGGGACGGCTGCCTTCGGGGGGGACGGGGCAGGGCGGGGTTCGGCTTCTGGCGTGTGACCGGCGGCTCTAGCCTCTGCTAACCAT<br>GTTCATGCCTTCTTCTTTTTCCTACAGCTCCTGGGCAACGTGCTGGTTGTTGTGCTGTCTCATCATTTTGGCAAATCTAGAGCCGCCACCATGCGGCTGGGGCTCCTGAGCGTGGCG<br>CTGTTGTTTGTGGGGAGCTCTCACTTATACTCAGACCACTACTCGCCCTCTGGAAGGCACAGGCTCGGCCCCTCGCCGGAACCGGCGGCTAGTTCCCAGCAGGCTGAGGCCGTCCGC<br>AAGAGGCTCCGGCGGCGGAGGGAGGGAGGGGCGCATGCAGAGGATTGTGGAACAGCACCGCTTAAGGATGTGTTGCAAGGGTCTCGGATTATAGGGGGCACCGAAGCACAAGCTGGC<br>GCATGGCCGTGGGTGGTGAGCCTGCAGATTAAATATGGCCGTGTTCTTGTTCATGTATGTGGGGGAACCCTAGTGAGAGAGAGGTGGGTCCTCACAGCTGCCCACTGCACTAAAGAC<br>GCTAGCGATCCTTTAATGTGGACAGCTGTGATTGGAACTAATAATATACATGGACGCTATCCTCATACCAAGAAGATAAAAATTAAAGCAATCATTATTCATCCAAACTTCATTTTG<br>GAATCTTATGTAAATGATATTGCACTTTTTCACTTAAAAAAAGCAGTGAGGTATAATGACTATATTCAGCCTATTTGCCTACCTTTTGATGTTTTCCAAATCCTGGACGGAAACACA<br>AAGTGTTTTATAAGTGGCTGGGGAAGAACAAAAGAAGAAGGTAACGCTACAAATATTTTACAAGATGCAGAAGTGCATTATATTTCTCGAAAGATGTGTAATTCTGAGAGGAGTTAT<br>GGGGGAATAATTCCTAACACTTCATTTTGTGCAGGTGATGAAGATGGAGCTTTTGATACTTGCAGGGGTGACAGTGGGGGACCATTAATGTGCTACTTACCAGAATATAAAAGATTT<br>TTTGTAATGGGAATTACCAGTTACGGACATGGCTGTGGTCGAAGAGGTTTTCCTGGTGTCTATATTGGGCCATCCTTCTACCAAAAGTGGCTGACAGAGCATTTCTTCCATGCAAGC<br>ACTCAAGGCATACTTACTATAAATATTTTACGTGGCCAGATCCTCATAGCTTTATGTTTTGTCATCTTACTAGCAACAACAGGCGGAGGCGGGTCCGGAGGCGGGGGCAGCGGCGGG<br>GGCGGAAGCGGGGGCGGAGGCTCCGGAGGCGGAGGGAGCGACTACAAAGACCATGACGGTGATTATAAAGATCATGACATTGATTACAAGGATGACGATGACAAGTAA

Figure S8. Transgenic mouse constructs in pCAG vector

Sequence begins at the CMV enhancer in the pCAG vector, restrictions sites used to ligate the protein into the plasmid were XhoI and XbaI

a. pCAG_PRSS55_3XFLAG_GPI

b. pCAG_PRSS55_TM_3XFLAG

c. pCAG_TMPRSS12_3XFLAG

Fig S9. Transgenic mouse constructs in pClgn vector.

**a.**	pCLGN_PRSS55_3XFLAG_GPI	TTGAGCGGGCCGCTTGCGCACTGGGGCTGCCACTGCATCCTGCATCCTCCGGCGCGGCCGCTCGCGCACGCGCAGTTAGCAGTCGCTGCTGGGCGGCTGCCGGCGGGATCGCTCTGG<br>GGAGACGCCGGGCACCAAGTAGCGGCCAGTCGGACACCTGAGCTGCCGCTGCTGGACACAAGCCCGCAGGACAGGTGTGTAGACCGCGGGCCGCCTTCCTGCGGCTTGTTCTCTGGG<br>GACCGGGTGAGAAATCGGATCtctagaGCCGCCACCATGCTCCTGTTCTCAGTGTTGCTGCTCCTGTCCCTGGTCACGGGAACTCAGCTCGGTCCACGGACTCCTCTCCCAGAGGCT<br>GGAGTGGCTATCCTAGGCAGGGCTAGGGGAGCCCACCGCCCTCAGCCCCCTCATCCCCCCAGCCCAGTCAGTGAATGTGGTGACAGATCTATTTTCGAGGGAAGAACTCGGTATTCC<br>AGAATCACAGGGGGGATGGAGGCGGAGGTGGGTGAGTTTCCGTGGCAGGTGAGTATTCAGGCAAGAAGTGAACCTTTCTGTGGCGGCTCCATCCTCAACAAGTGGTGGATTCTCACT<br>GCGGCTCACTGCTTATATTCCGAGGAGCTGTTTCCAGAAGAACTGAGTGTCGTGCTGGGGACCAACGACTTAACTAGCCCATCCATGGAAATAAAGGAGGTCGCCAGCATCATTCTT<br>CACAAAGACTTTAAGAGAGCCAACATGGACAATGACATTGCCTTGCTGCTGCTGGCTTCGCCCATCAAGCTCGATGACCTGAAGGTGCCCATCTGCCTCCCCACGCAGCCCGGCCCT<br>GCCACATGGCGCGAATGCTGGGTGGCAGGTTGGGGCCAGACCAATGCTGCTGACAAAAACTCTGTGAAAACGGATCTGATGAAAGCGCCAATGGTCATCATGGACTGGGAGGAGTGT<br>TCAAAGATGTTTCCAAAACTTACCAAAAATATGCTGTGTGCCGGATACAAGAATGAGAGCTATGATGCCTGCAAGGGTGACAGTGGGGGGCCTCTGGTCTGCACCCCAGAGCCTGGT<br>GAGAAGTGGTACCAGGTGGGCATCATAAGCTGGGGAAAGAGCTGTGGAGAGAAGAACACCCCAGGGATATACACCTCGTTGGTGAACTACAACCTCTGGATCGAGAAAGTGACCCAG<br>CTAGAGGGCAGGCCCTTCAATGCAGAGAAAAGGAGGACTTCTGTCAAACAGAAACCTATGGGCGGCGGAGGCGGGTCCGACTACAAAGACCATGACGGTGATTATAAAGATCATGAC<br>ATTGATTACAAGGATGACGATGACAAGGGCGGAGGCGGGTCCTCCCCAGTCTCGGGAGTCCCAGAGCCAGGCAGCCCCAGATCCTGGCTCCTGCTCTGTCCCCTGTCCCATGTGTTG<br>TTCAGAGCTATTTTGTACTGA
**b.**	pCLGN_PRSS55_TM_3XFLAG	TTGAGCGGGCCGCTTGCGCACTGGGGCTGCCACTGCATCCTGCATCCTCCGGCGCGGCCGCTCGCGCACGCGCAGTTAGCAGTCGCTGCTGGGCGGCTGCCGGCGGGATCGCTCTGG<br>GGAGACGCCGGGCACCAAGTAGCGGCCAGTCGGACACCTGAGCTGCCGCTGCTGGACACAAGCCCGCAGGACAGGTGTGTAGACCGCGGGCCGCCTTCCTGCGGCTTGTTCTCTGGG<br>GACCGGGTGAGAAATCGGATCtctagaGCCGCCACCATGCTCCTGTTCTCAGTGTTGCTGCTCCTGTCCCTGGTCACGGGAACTCAGCTCGGTCCACGGACTCCTCTCCCAGAGGCT<br>GGAGTGGCTATCCTAGGCAGGGCTAGGGGAGCCCACCGCCCTCAGCCCCCTCATCCCCCCAGCCCAGTCAGTGAATGTGGTGACAGATCTATTTTCGAGGGAAGAACTCGGTATTCC<br>AGAATCACAGGGGGGATGGAGGCGGAGGTGGGTGAGTTTCCGTGGCAGGTGAGTATTCAGGCAAGAAGTGAACCTTTCTGTGGCGGCTCCATCCTCAACAAGTGGTGGATTCTCACT<br>GCGGCTCACTGCTTATATTCCGAGGAGCTGTTTCCAGAAGAACTGAGTGTCGTGCTGGGGACCAACGACTTAACTAGCCCATCCATGGAAATAAAGGAGGTCGCCAGCATCATTCTT<br>CACAAAGACTTTAAGAGAGCCAACATGGACAATGACATTGCCTTGCTGCTGCTGGCTTCGCCCATCAAGCTCGATGACCTGAAGGTGCCCATCTGCCTCCCCACGCAGCCCGGCCCT<br>GCCACATGGCGCGAATGCTGGGTGGCAGGTTGGGGCCAGACCAATGCTGCTGACAAAAACTCTGTGAAAACGGATCTGATGAAAGCGCCAATGGTCATCATGGACTGGGAGGAGTGT<br>TCAAAGATGTTTCCAAAACTTACCAAAAATATGCTGTGTGCCGGATACAAGAATGAGAGCTATGATGCCTGCAAGGGTGACAGTGGGGGGCCTCTGGTCTGCACCCCAGAGCCTGGT<br>GAGAAGTGGTACCAGGTGGGCATCATAAGCTGGGGAAAGAGCTGTGGAGAGAAGAACACCCCAGGGATATACACCTCGTTGGTGAACTACAACCTCTGGATCGAGAAAGTGACCCAG<br>CTAGAGGGCAGGCCCTTCAATGCAGAGAAAAGGAGGACTTCTGTCAAACAGAAACCTATGGGCTCCCCAGTCATACTTACTATAAATATTTTACGTGGCCAGATCCTCATAGCTTTA<br>TGTTTTGTCATCTTACTAGCAACAACAGGCGGAGGCGGGTCCGGAGGCGGGGGCAGCGGCGGGGGCGGAAGCGGGGGCGGAGGCTCCGGAGGCGGAGGGAGCGACTACAAAGACCAT<br>GACGGTGATTATAAAGATCATGACATTGATTACAAGGATGACGATGACAAGTGA
**c.**	pCLGN_TMPRSS12_3XFLAG	TTGAGCGGGCCGCTTGCGCACTGGGGCTGCCACTGCATCCTGCATCCTCCGGCGCGGCCGCTCGCGCACGCGCAGTTAGCAGTCGCTGCTGGGCGGCTGCCGGCGGGATCGCTCTGG<br>GGAGACGCCGGGCACCAAGTAGCGGCCAGTCGGACACCTGAGCTGCCGCTGCTGGACACAAGCCCGCAGGACAGGTGTGTAGACCGCGGGCCGCCTTCCTGCGGCTTGTTCTCTGGG<br>GACCGGGTGAGAAATCGGATCtctagaGCCGCCACCATGCGGCTGGGGCTCCTGAGCGTGGCGCTGTTGTTTGTGGGGAGCTCTCACTTATACTCAGACCACTACTCGCCCTCTGGA<br>AGGCACAGGCTCGGCCCCTCGCCGGAACCGGCGGCTAGTTCCCAGCAGGCTGAGGCCGTCCGCAAGAGGCTCCGGCGGCGGAGGGAGGGAGGGGCGCATGCAGAGGATTGTGGAACA<br>GCACCGCTTAAGGATGTGTTGCAAGGGTCTCGGATTATAGGGGGCACCGAAGCACAAGCTGGCGCATGGCCGTGGGTGGTGAGCCTGCAGATTAAATATGGCCGTGTTCTTGTTCAT<br>GTATGTGGGGGAACCCTAGTGAGAGAGAGGTGGGTCCTCACAGCTGCCCACTGCACTAAAGACGCTAGCGATCCTTTAATGTGGACAGCTGTGATTGGAACTAATAATATACATGGA<br>CGCTATCCTCATACCAAGAAGATAAAAATTAAAGCAATCATTATTCATCCAAACTTCATTTTGGAATCTTATGTAAATGATATTGCACTTTTTCACTTAAAAAAAGCAGTGAGGTAT<br>AATGACTATATTCAGCCTATTTGCCTACCTTTTGATGTTTTCCAAATCCTGGACGGAAACACAAAGTGTTTTATAAGTGGCTGGGGAAGAACAAAAGAAGAAGGTAACGCTACAAAT<br>ATTTTACAAGATGCAGAAGTGCATTATATTTCTCGAAAGATGTGTAATTCTGAGAGGAGTTATGGGGGAATAATTCCTAACACTTCATTTTGTGCAGGTGATGAAGATGGAGCTTTT<br>GATACTTGCAGGGGTGACAGTGGGGGACCATTAATGTGCTACTTACCAGAATATAAAAGATTTTTTGTAATGGGAATTACCAGTTACGGACATGGCTGTGGTCGAAGAGGTTTTCCT<br>GGTGTCTATATTGGGCCATCCTTCTACCAAAAGTGGCTGACAGAGCATTTCTTCCATGCAAGCACTCAAGGCATACTTACTATAAATATTTTACGTGGCCAGATCCTCATAGCTTTA<br>TGTTTTGTCATCTTACTAGCAACAACAGGCGGAGGCGGGTCCGGAGGCGGGGGCAGCGGCGGGGGCGGAAGCGGGGGCGGAGGCTCCGGAGGCGGAGGGAGCGACTACAAAGACCAT<br>GACGGTGATTATAAAGATCATGACATTGATTACAAGGATGACGATGACAAGTAA

Figure S9. Transgenic mouse constructs in pClgn vector

Sequence begins at the Cgln promoter in the pCgln vector, restrictions sites used to ligate the protein into the plasmid were XhoI and XbaI

a. pClgn_PRSS55_3XFLAG_GPI

b. pClgn_PRSS55_TM_3XFLAG

c. pClgn_TMPRSS12_3XFLAG
